# Mapping the primate thalamus: historical perspective and modern approaches for defining nuclei

**DOI:** 10.1007/s00429-022-02598-4

**Published:** 2023-01-09

**Authors:** Miguel Ángel García-Cabezas, Isabel Pérez-Santos, Carmen Cavada

**Affiliations:** 1 Department of Anatomy, Histology and Neuroscience, School of Medicine, Universidad Autónoma de Madrid, Calle Arzobispo Morcillo 4, 28029 Madrid, Spain; 2 PhD Program in Neuroscience, Universidad Autónoma de Madrid-Cajal, Madrid, Spain; 3 Neural Systems Laboratory, Department of Health Sciences, Boston University, Boston, MA USA

**Keywords:** Thalamic nucleus, Nuclear parcellation, Cytoarchitecture, Myeloarchitecture, Genoarchitecture, New Neuromorphology

## Abstract

The primate thalamus has been subdivided into multiple nuclei and nuclear groups based on cytoarchitectonic, myeloarchitectonic, connectional, histochemical, and genoarchitectonic differences. Regarding parcellation and terminology, two main schools prevailed in the twentieth century: the German and the Anglo-American Schools, which proposed rather different schemes. The German parcellation and terminology has been mostly used for the human thalamus in neurosurgery atlases; the Anglo-American parcellation and terminology is the most used in experimental research on the primate thalamus. In this article, we review the historical development of terminological and parcellation schemes for the primate thalamus over the last 200 years. We trace the technological innovations and conceptual advances in thalamic research that underlie each parcellation, from the use of magnifying lenses to contemporary genoarchitectonic stains during ontogeny. We also discuss the advantages, disadvantages, and practical use of each parcellation.

## Introduction

The thalamus is a mass of subcortical gray matter on the lateral walls of the third ventricle. Some thalamic neurons relay sensory information from the periphery to the cerebral cortex and others participate in cognitive and emotional processing through strong reciprocal connections with the cerebral cortex and connections with the amygdala, striatum, and hypothalamus (Jones 2007).

The structure of the thalamus is not homogeneous. Thalamic neurons are diverse and, together with glial cells, are distributed unevenly across multiple nuclei that can be identified and delineated by microscopic examination of brain sections stained with different techniques. Neuroanatomists of the last 200 years identified multiple nuclei and nuclear groups in the primate thalamus based on cytoarchitectonic, myeloarchitectonic, connectional, and histochemical differences that prompted different theoretical paradigms. Regarding terminology, two main schools, the German and the Anglo-American Schools, prevailed in the twentieth century and proposed rather different schemes for primate thalamic nuclei. The German terminology is mostly used in atlases of human neurosurgery (e.g., Schaltenbrand and Bailey 1959; Andrew and Watkins 1968; Van Buren and Borke 1972; Tasker 1990). In contrast, the Anglo-American terminology is mostly used in experimental research on primates, including humans (Hirai and Jones 1989) and non-human primates (e.g., Olszewski 1952; Goldman-Rakic and Porrino 1985; Sánchez-González et al. 2005). More recently, genoarchitectonic studies using in situ hybridization in vertebrate embryos to detect the spatial and temporal expression of morphogenetic genes across the neural plate and neural tube have opened the path for a New Neuromorphology (Nieuwenhuys and Puelles 2016), which is based on the origin of brain structures in ontogeny and phylogeny, and will likely impact future parcellations and terminologies for thalamic nuclei in human and non-human primates.

The topic of thalamic nuclear parcellation and terminology has captured and still captures the interest of clinical and basic neuroscientists, as shown by recent publications on the history of thalamic investigations (Serra et al. 2019; Clascá 2022). Some researchers have specifically focused on the problems posed by the lack of a common terminology for thalamic nuclei in primates and have proposed equivalences between the various available terminologies (Axer and Niemann 1994; Macchi and Jones 1997). A commonly agreed upon terminology for the nuclei of the primate thalamus would help advance thalamic research for two major reasons. First, it would facilitate the comparison of experimental data obtained from studies in non-human primates. Second, it would favor communication between neuroscientists, neurologists, and neurosurgeons operating on the human thalamus. In this scenario, a recent review has proposed a novel approach that evades classical terms and nuclear divisions (Mai and Majtanik 2018). The authors of that review propose converting the terminology for thalamic nuclei into spatial coordinates to facilitate communication between researchers and clinicians.

In the present article, we intend to contribute to the ongoing debate on thalamic terminology by reviewing the historical development of the terminology for thalamic nuclei in human and non-human primates from the early nineteenth century to the present time. We trace the technological innovations and conceptual advances in neuroscientific research that underlie each nuclear parcellation and terminology referring to the primate thalamus over the last 200 years. Our goals are to investigate the epistemological bases of each parcellation and discuss the advantages, disadvantages, and practical uses for each parcellation and its corresponding terminology.

## First parcellations of the human thalamus in Germany and France (nineteenth century)

The first descriptions of thalamic nuclei in the human thalamus were carried out by neurologists and psychiatrists in Germany and France during the nineteenth century. These early studies of thalamic structure were done with simple methods whose progressive elaboration (from unstained tissue to a variety of tissue stains, from magnifying lenses to compound optical microscopes with apochromatic objectives) allowed for basic thalamic parcellations that prepared the field for further experimental approaches. Early thalamic parcellations and nuclear terminologies of the human thalamus (Table 1) and the technological advances that made them possible are explained below.

**Table 1:** Early nuclear parcellations of the human and non-human primate thalamus

Burdach (1822)	Luys (1865)	Forel (1877)	von Monakow (1895)	von Kölliker (1896)	Sachs (1909)
Inner	Centre moyen	Inner	Medial group: nuclei med. a, med. b (médian center of Luys), and med. c	Mediale Kern (Nml)	Nucleus medius (NM, MN)
	Centre médian	Centre médian		Mittlere Kern-Centre médian of Luys (Nm)	Centre median (CM)
Äussere	Région externe	Äussere	Lateral group: nuclei lat. a and lat. b	Laterale Kern: Lateral nucleus (Nl)	Nucleus lateralis (NL) (dorsal portion, middle portion, ventral portion)
			Ventral group: vent. ant, vent. a, vent. b, and vent. c		Nucleus ventralis (VN, NV) Nucleus arcuatus (NA)
Obre	Centre antérieur	Ober	Anterior group: nuclei ant. a, ant. b, and ant. c	Dorsale Kern (Nd)	Nucleus anterior (AN)
Polster	Centre postérieur	Pulvinar	Pulvinar	Pulvinar	Pulvinar (Pu)
–	–	–	Posterior group (a part of the current posterior group)	–	–
Corpora geniculata	Corps genouillés	Corpora geniculata	Corpus geniculatum internum	Corpus geniculatum mediale	–
			Corpus geniculatum externum	Corpus geniculatum laterale	
Hornblatt-lamina cornea	–	Lamina medullaris externa and Gitterschicht	Striped or grilled group (reticular nucleus and lateral medullary lamina)	Gitterschicht	–
				Lamina medullaris externa	

The first researcher of brain structure who clearly recognized nuclei in the human thalamus was the German neurologist Karl Friedrich Burdach (1776–1847). Burdach used magnifying glasses to examine blocks of human brain fixed with alcohol (Meyer 1970). With this simple technology he identified the internal medullary lamina (*Das Marckblatt des Sehhügels-lamina medullaris thalami*) and defined three nuclei in relation to that lamina: (1) *Inner grane Kern*-*nucleus cinereus internus*, inner nucleus; (2) *Äussere grane Kern*-*nucleus cinereus externus*, outer nucleus; (3) *Obre grane Kern*-*nucleus cinereus superior*, upper nucleus. Burdach also identified the pulvinar (*Polster*) nucleus, the geniculate nuclei (*Äussere Kniehöcker-corpus geniculatum externus*, lateral geniculate nucleus*; inner Kniehöcker-corpus geniculatum internum*, medial geniculate nucleus), and a *lamina cornea* (*Hornblatt*) that probably consisted of the external medullary lamina and the reticular nucleus of the thalamus (Table 1) (Burdach 1822).

**Fig. 1:**
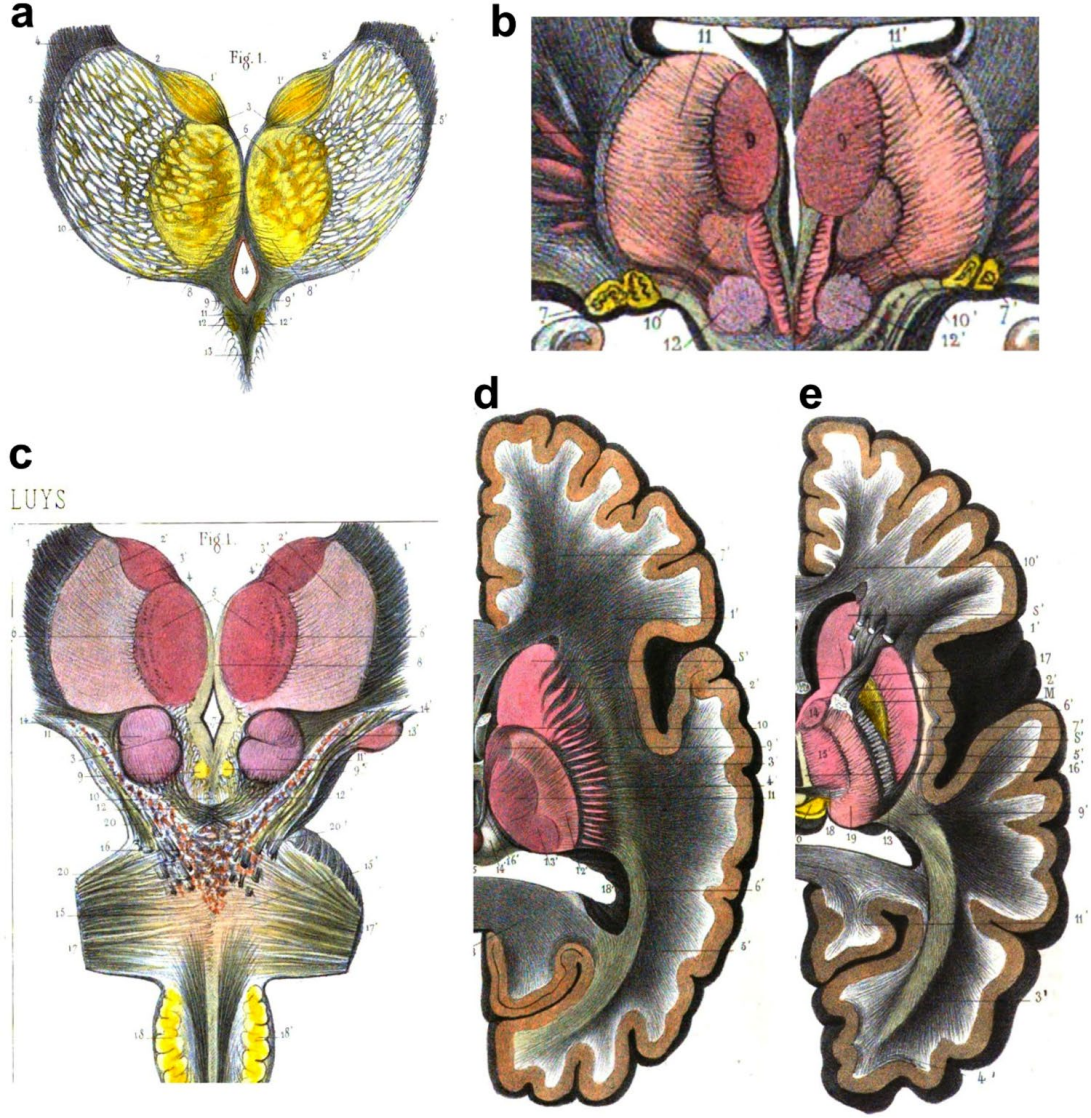
Drawings including the human thalamus and related tracts of fibers, from *“Recherches sur le Système Nerveux Cérébro-spinal: Sa Structure, ses Fonctions et ses Maladies”,* Luys (1865). **a**–**c** Drawings of coronal brain sections depicting the thalamus, including the following nuclei: the *centres antérieurs* (**a**-1,1′; **c**-3,3′), the *centres moyens* (**a**-6; **b**-9; **c**-5), the *centres médians* (**b**-10,10′), the *région externe des couches optiques* (**a**-5,5′; **b**-11,11′; **c**-2,2′), and the *corps genouillés* (**b**-7,7′). **d**, **e** Drawings of a brain hemisphere cut in the horizontal plane containing the thalamus (left is medial, right is lateral, top is anterior, bottom is posterior); **d** is superior, **e** is inferior. The following nuclei are depicted: the *centres antérieurs* (**d**-9′; **e**-14), the *centres moyens* (**d**-11; **e**-15′), the *centres postérieurs* (**d**-13′; **e**-19), and the *région externe des couches optiques* (**d**-12′). Reproduced from the digitized exemplar of “*Recherches* *sur* *le* *Système* *Nerveux*…” in the *Bibliothèque* *municipale de* *Lyon* (https://numelyo.bm-lyon.fr/f_view/BML:BML_00GOO0100137001102623480)

When Burdach carried out his research on the human thalamus he did not have the technologies needed for tissue processing and microscopic analysis, such as tissue hardeners and fixatives, inclusion media, microtomes for cutting series of thin slices of brains, stains for nervous tissue, and compound optical microscopes with lenses to correct optical aberrations. All these technologies were developed between 1830 and 1890 and allowed the histological observations across tissues and species that founded the Cell Theory and culminated in the Neuron Theory (Bracegirdle 1978; Shepherd 1991; Hakosalo 2006). Some of these technological innovations were at hand for the French neurologist Jules Bernard Luys (1828–1897), who was among the first scientists to examine the brain under the microscope (Parent 2002; Parent et al. 2002; Parent and Parent 2011). Luys cut 1 cm-thick slabs of human brain and fixed and hardened them with a solution of 4% chromic acid in water; then, he cut thin slices from these blocks with a double knife and either rendered the slices transparent with glycerin or colored them with carmine red. Transparent sections allowed identification of myelinated bundles due to differences in the refraction index of myelinated versus non-myelinated tissue and provided basic myeloarchitectonic pictures; carmine red stained nuclei of neurons and glial cells, as well as neuron bodies, providing elementary cytoarchitectonic pictures (Luys 1865). Luys examined the microscopic structure of the human thalamus in transparent sections and in carmine red-stained sections and described four nuclei that he called centers: (1) *centre antérieur* (upper nucleus of Burdach), which processed olfactory sensations; (2) *centre moyen* (inner nucleus of Burdach), which processed visual information; (3) *centre médian*, which processed somatosensory information; and (4) *centre postérieur* (a part of the pulvinar nucleus), which processed auditory information (Table 1). The functions attributed to each thalamic nucleus were based on their connections with sensory structures by axon bundles whose origins, trajectories, and targets were identified by Luys in transparent sections. Luys also mentioned the medial and lateral geniculate nuclei (*corps genouillés*) and referred to the outer nucleus of Burdach as *région externe des couches optiques* (outer region of the optic thalamus), which he considered as just a passing place for fibers on their way to the other thalamic nuclei (Fig. 1).

Finally, he described convergent fibers concentrated “*au pourtour de la couche optique*” in the site of the *lamina cornea* of Burdach. All these observations led Luys to suggest that the thalamus was the common receptor of sensations:

*“La couche optique représente le centre commun, le récepteur unique, dans lequel la plupart des fibres du systéme convergent inférieur (émanées des divers plexus sensoriels périphériques) et la plupart des fibres du système convergent supérieur (émergées de la périphérie cérébrale) viennent successivement s'amortir et se combiner les unes avec les autres”*. [The optic thalamus represents the common center, the unique receptor, in which most inferior convergent system fibers (emanating from several peripheral sensory plexuses) and most of the superior convergent system fibers (emerging from the cerebral periphery) successively arrive to meet and combine with each other] [Luys (1865), p. 196].

The German psychiatrist Theodor Hermann Meynert (1833–1892) also examined the microscopic structure of the human brain in transparent sections and sections stained with carmine red (Hakosalo 2006; Triarhou 2021). Applying these techniques to the human thalamus, Meynert described the habenula and the habenulo-interpeduncular tract, now also called Meynert’s bundle. However, Meynert thought that nuclear parcellation was not justified in the thalamus:

“There is only a partial justification for admitting the existence within this general form of special nuclei in the interior of the optic thalami; for the whole of the gray matter of the optic thalamus forms a continuous mass, and no characteristic differences in its textual composition have hitherto been satisfactorily demonstrated”. [Meynert (1872), p. 426].

**Fig. 2:**
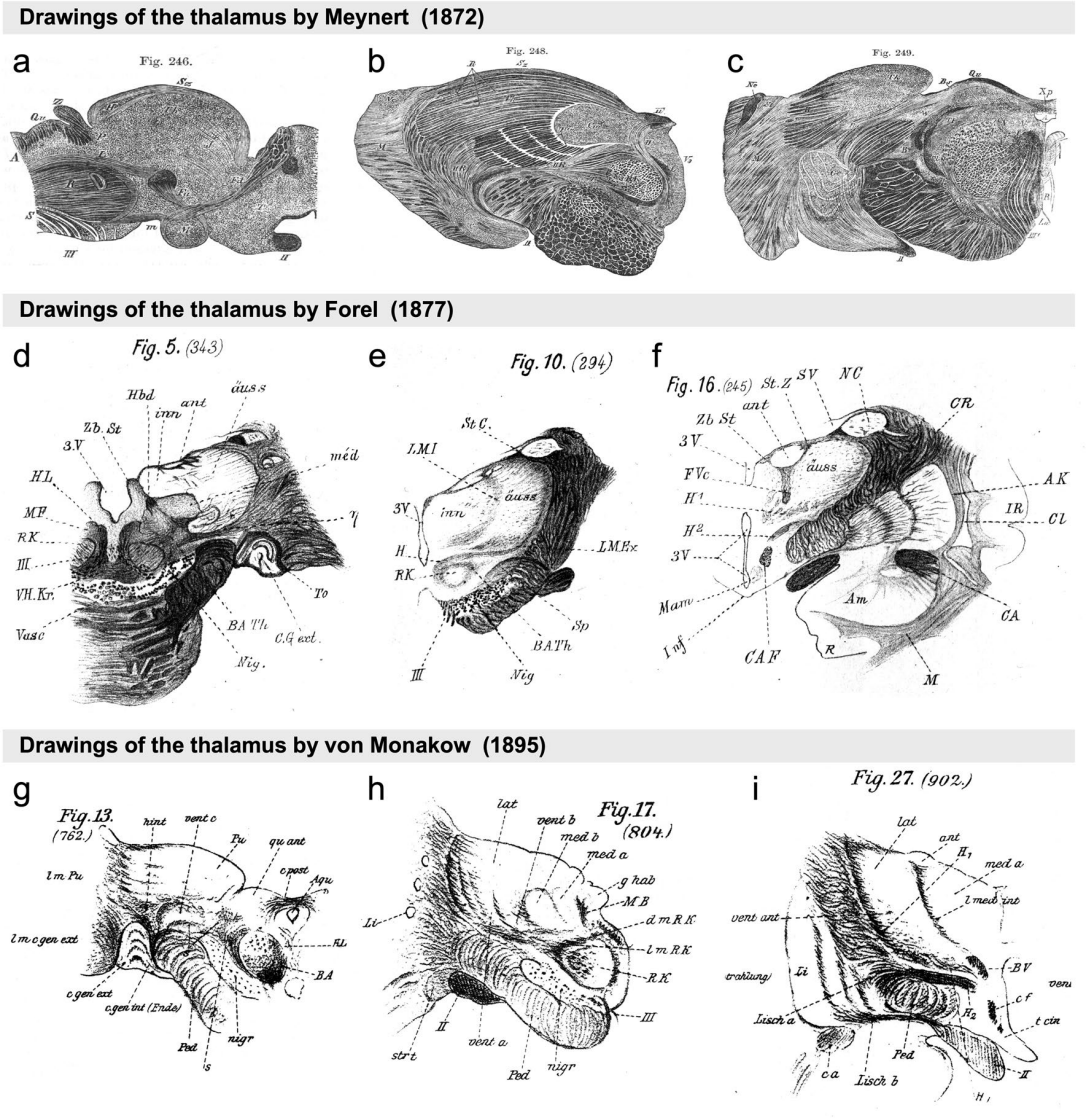
Drawings of the nuclear parcellation of the human thalamus by German authors in the second half of the nineteenth century. **a**–**c** Drawings made after sagittal (**a**) and coronal (**b**, **c**) transparent sections by Meynert (1872); the stain used shows poor differentiation of thalamic nuclei. **d**–**f** Drawings made from transparent coronal sections by Forel (1877). **g**–**i** Drawings made from sections stained with carmine red by von Monakow (1895). Images from Meynert (1872) are reproduced from a copy of the original publication in the private collection of Dr. García-Cabezas. Images from Forel (1877) and von Monakow (1895) are reproduced from copies of the *Archiv für Psychiatrie und Nervenkrankheiten* journal in the Library of Cajal Institute (Spanish Research Council-CSIC-Library Network), Madrid, Spain

Accordingly, Meynert did not highlight nuclear divisions and boundaries in his illustrations of the human thalamus (Fig. 2a–c). In contrast, the Swiss psychiatrist Auguste Forel (1848–1931), who undertook his doctoral thesis on the human thalamus under the direction of Meynert (Akert 1993), parcellated the human thalamus into nuclei (Fig. 2d–f) and provided a synthesis of the parcellations and terminologies of Burdach and Luys (Table 1). Forel used the microtome invented in 1875 by the German psychiatrist Bernhard Aloys von Gudden (1824–1886) to obtain serial sections through the human thalamus and stained them with carmine red and chromic acid, obtaining a mixed picture of cytoarchitecture and myeloarchitecture (Forel 1877; Hakosalo 2006). Using Burdach’s parcellation scheme, Forel distinguished the external medullary lamina and the reticular nucleus, which he called *Gitterschicht* (lattice nucleus), and added the *centre médian* of Luys:

*“Nach Burdach (a. a. O. Bd. II. S. 121) besteht der Thalamus ausser dem Pulvinar (Pulv. in unseren Fig.) aus drei grauen Kernen: 1) innerer Kern (inn. in unseren Fig.), 2) äusserer Kern (äuss.), 3) oberer Kern (ant.). Als Lamina medullaris Thalami bezeichnet er ein Markblatt, das den inneren Kern vom äusseren trennt (LMI, Fig. 8*, 10*), als Lamina cornea die Lamina medullaris externa(?) oder die Gitterschicht(?). Diese Darstellung Burdach's ist eine nahezu ganz tadellose, und daher ist sie beizubehalten, oder, besser gesagt, wieder zu Ehren zu bringen”*. [Burdach described three nuclei, apart from the pulvinar nucleus: 1) inner nucleus (inn in our Figure); 2) outer nucleus (äuss); and 3) upper nucleus (ant). As *Lamina medullaris thalami* he referred to a mark separating the inner from the outer nucleus (LMI, Fig. 8, 10). As *Lamina cornea* he designated the *Lamina medullaris externa* (?) or striped (?). Burdach’s division is virtually impeccable therefore it should be retained, or rather, its prestige should be re-established] [Forel (1877), p. 483].

In summary, the first period of early anatomical studies of the human thalamus begins with Burdach’s investigations and concludes with the parcellation and terminology of thalamic nuclei of August Forel, which integrates Luys’s contribution. Forel’s parcellation was still crude and lacked fundamental connectional data. Finer nuclear divisions would arrive with better tissue stains and data on connections of thalamic nuclei with the cerebral cortex that were obtained experimentally in research animals in the following decades by neuroanatomists in German-speaking countries.

## Origin of the German School of thalamic studies: Constantin von Monakow

The most used stain for microscopic studies of the brain and other tissues between 1860 and 1880 was carmine red. But from 1870 on, rapid innovations took place in the laboratories of histology across Europe. Significantly, novel techniques were developed for staining myelin and nerve cells, as well as for tracing connections both retrogradely and anterogradely based on degeneration secondary to lesion (Farrar 1905; Bracegirdle 1978; Shepherd 1991; Hakosalo 2006). These technological innovations facilitated systematic microscopic studies on the mammalian thalamus and led in German-speaking Europe to the development of a school of thalamic researchers that we will call the German School.

The initiator of the German School of thalamic studies was Constantin von Monakow (1853–1930), a Russian-born neurologist who settled in Zurich. Von Monakow used carmine red stain to survey the entire thalamus of several mammalian species, including humans, and sorted thalamic nuclei into nine groups (von Monakow 1895):

I. Anterior group: nuclei ant. a, ant. b, and ant. c.
II. Medial group: nuclei med. a, med. b (median center of Luys), and med. c.
III. Lateral group: nuclei lat. a and lat. b.
IV. Ventral group: vent. ant, vent. a, vent. b, and vent. c.
V. Posterior group (a part of the current posterior group).
VI. Striped or grilled group (reticular nucleus and lateral medullary lamina).
VII. Pulvinar.
VIII. Lateral geniculate body.
IX. Medial geniculate body (Table 1).

**Fig. 3:**
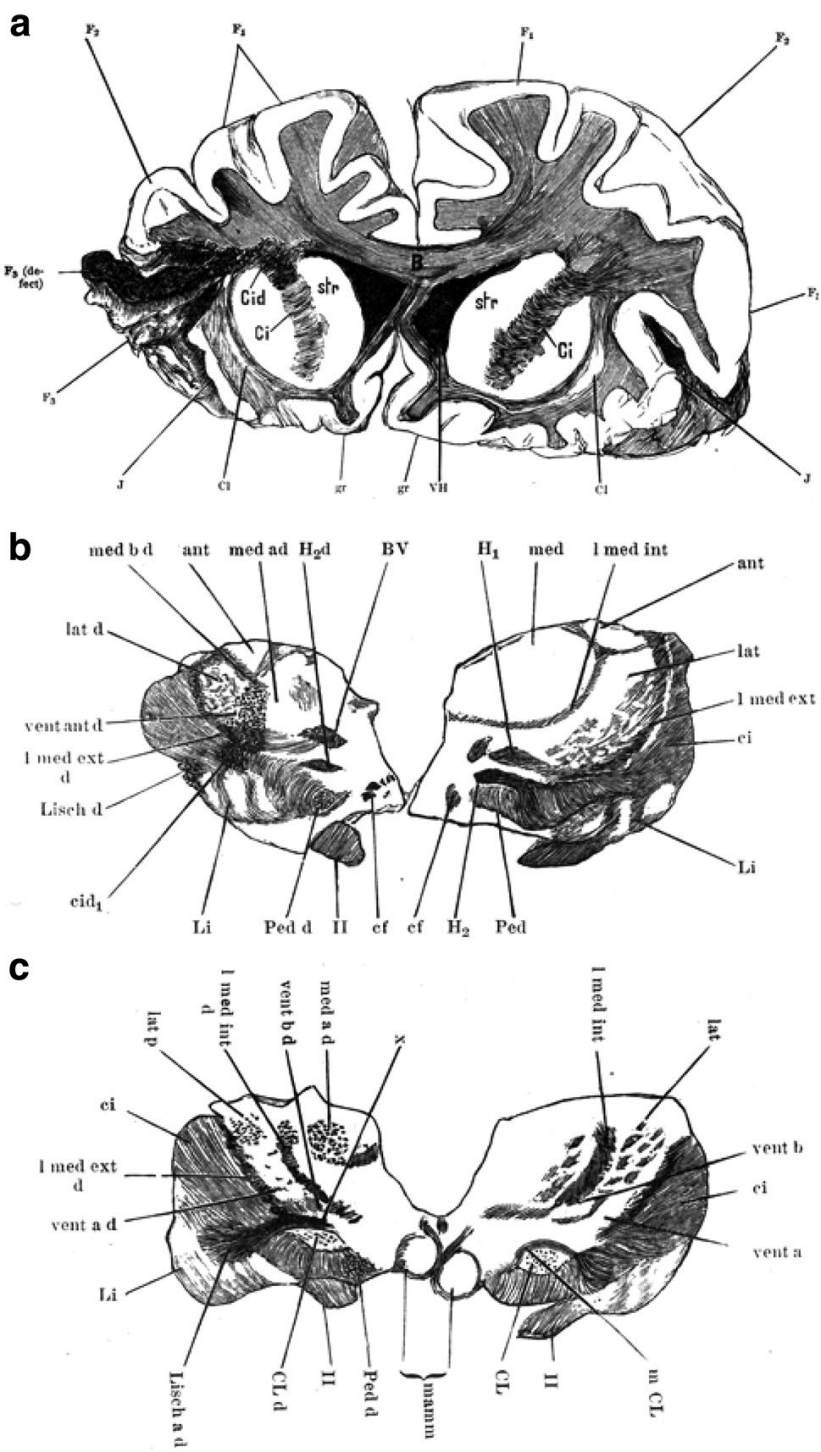
Drawings of a postmortem human brain showing retrograde degeneration by von Monakow (1895). **a** Coronal section of the frontal lobes with a lesion involving the left orbital and anterior insular cortex. **b**, **c** Drawings of coronal sections through the thalamus showing retrograde degeneration in thalamic nuclei secondary to the cortical lesion depicted in (**a**). Images are reproduced from a copy of the *Archiv für Psychiatrie und Nervenkrankheiten* journal in the library of Cajal Institute (Spanish Research Council-CSIC-Library Network), Madrid, Spain

Thus, von Monakow classified thalamic nuclei into groups that are still used, except for the intralaminar and midline groups, which are terms that von Monakow never used (Fig. 2g–i). Along with nuclear parcellation, von Monakow studied thalamo-cortical connections using Gudden’s technique in dogs, cats, and humans with cortical lesions (von Monakow 1895). The Gudden technique consists of identifying retrograde degeneration in neuronal bodies secondary to surgical lesions in the brains of newborn animals. Briefly, after producing a surgical lesion, the animal is killed and its brain extracted, fixed, cut, and stained with cell-staining techniques, such as carmine red; then, researchers examine the carmine red-stained sections with optical microscopes looking for signs of retrograde neuronal degeneration; the parts of the brain whose neurons show signs of this degeneration are assumed to project to the injured areas (von Gudden 1870; LaVail 1975). In postmortem studies of human subjects, von Monakow could correlate lesions in the cerebral cortex with secondary degeneration in thalamic nuclei (Fig. 3).

In the cerebral cortex of dogs and cats, von Monakow produced extensive, though poorly localized, surgical lesions with imprecise results. However, he was the first to systematically address thalamocortical connections and his experiments were the key reference for all works in the field for a long time. Thus, von Monakow is to be considered the neuroanatomist who, at the turn of the twentieth century, best systematized the parcellation and thalamocortical connections of the mammalian thalamus.

**Fig. 4:**
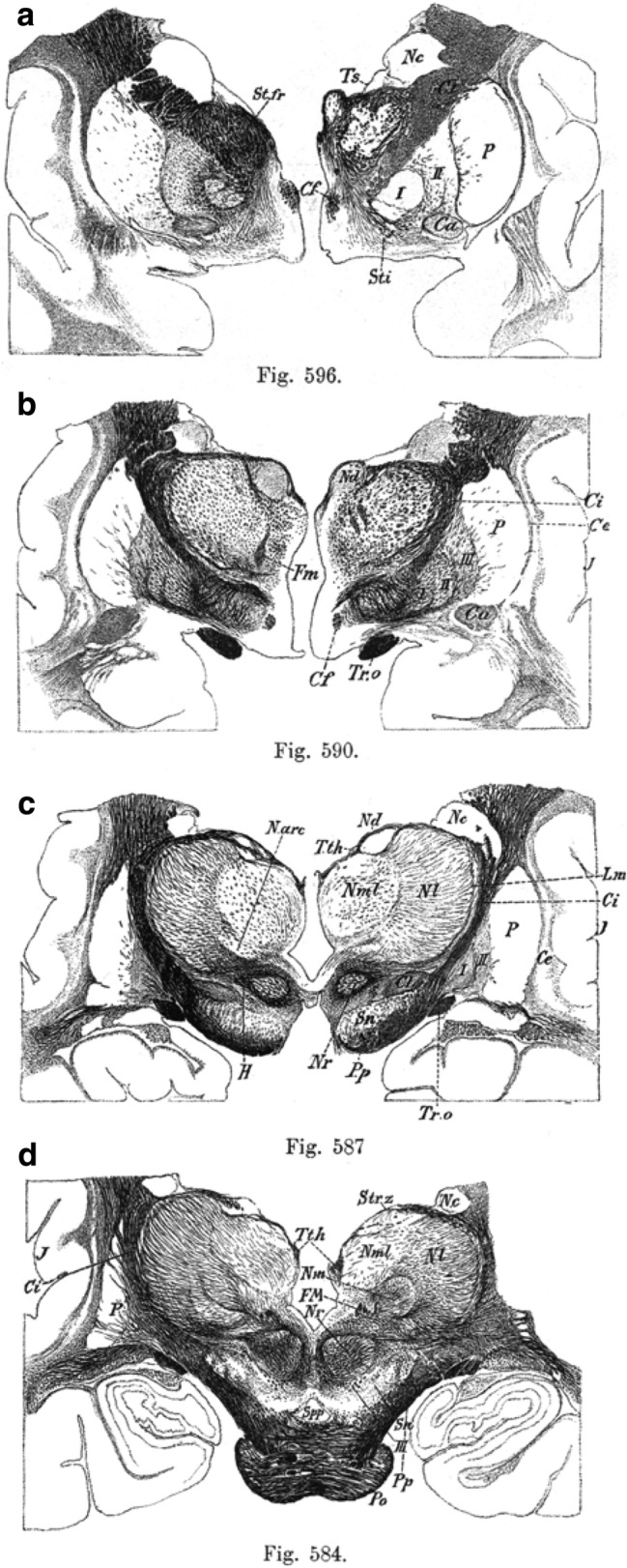
Drawings of coronal human brain sections depicting the thalamus from rostral (**a**) to caudal (**d**) by von Kölliker (1896), based on Nissl’s terminology (Nissl 1889). Images are reproduced from a copy of the original publication in the private collection of Dr. García-Cabezas

The parcellation and nuclear terminology of the human thalamus proposed by von Monakow were partially accepted by Rudolph Albert von Kölliker (1817–1905), Chairman of the German Society of Anatomy. Kölliker, in the sixth edition of his influential “*Handbuch der Gewebelehre des Menschen*” (von Kölliker 1896), described the two medullary laminae and the following nuclei in the human thalamus (Fig. 4):

1. Dorsale Kern (Nd).
2. Mediale Kern (Nml).
3. Mittlere Kern-Centre médian of Luys (Nm).
4. Laterale Kern: Lateral nucleus (Nl).
5. Gitterschicht-Reticular nucleus.
6. Pulvinar.
7. Corpus geniculatum laterale.
8. Corpus geniculatum mediale (Table 1).

Years later, Cécile Vogt would state:

*“C'est à Mr. von Monakow*^*2*^*) que revient le mérite d'avoir publié, le premier, une division plus détaillée de la couche optique des carnivores et de l'homme. Il se basait sur des différences histologiques (surtout cytoarchitecturales) et fibrosystématiques. L'accueil défavorable que Koelliker*^*3*^*) fit à son essai n'était pas du tout justifié”.* [It is Mr. Monakow who has the merit of having first published a detailed division of the human and carnivore thalamus. He was based on histological (mostly cytoarchitectonic) and fibrosystematic differences. The unfavorable opinion that Kolliker expressed in his essay is not at all justified] [Vogt (1909), p. 286].

Kölliker used the technique developed by the German pathologist Karl Weigert (1845–1904) to stain myelin. The Weigert technique consists in treating nervous tissue with potassium dichromate to preserve myelin lipids followed by copper incubation; then lipids are stained with hematoxylin (Weigert 1884). This technique provided much finer pictures of myeloarchitecture (Fig. 4) than the primitive transparent sections used by Luys and Meynert and the double-staining of carmine red and chromic acid used by Forel. Kölliker also showed the nuclear parcellation of the rabbit thalamus according to the German neurohistologist Franz Nissl (1869–1919). Nissl dyed brain tissue with toluidine blue, a basic aniline with affinity for acidic structures of the cell such as nuclei and cytoplasmic ribosomes. Nissl technique, commonly used nowadays in most neuroscience laboratories, stains the nuclei of all neurons, glial cells, and endothelial cells as well as the body of neurons, leaving the neuropil unstained (García-Cabezas et al. 2016).

Compared to carmine red, Nissl staining provided better and sharper cytoarchitectonic pictures that allowed Nissl himself to identify distinct thalamic nuclei divided by sharp boundaries (Nissl 1889). Nissl subdivided the anterior and medial nuclei into three subnuclei each, and the lateral and pulvinar nuclei into two subnuclei each. He also used the term ventral to name a portion of the lateral nucleus and identified the midline nuclei (*Kern der Mittellinie*) for the first time. Nissl’s studies on the rabbit thalamus were first published without illustrations as a summary abstract for a paper presented in a scientific meeting (Nissl 1889). The definitive paper, with plenty of micrographs, was not published until 1913 (Nissl 1913). Nissl’s work on the thalamus was influential thanks to the publicity it received from von Kölliker (1896). For instance, the Spanish neurohistologist Santiago Ramón y Cajal (1852–1934), who studied the thalamus of small mammals with little reference to the human thalamus, used Nissl’s terminology, endorsed by his friend Kölliker. Nevertheless, occasionally, Cajal used new terms to refer to some nuclei, though he always added the term used by Nissl and Kölliker in brackets (Ramón y Cajal 1904). Cajal classified thalamic nuclei into three anteroposterior series: (1) the external series included, from back to front, the medial geniculate body or auditory ganglion, the lateral geniculate body or lower optic ganglion, the pulvinar nucleus or upper optic ganglion, and the striped ganglion (*Gitterkern*) of Nissl; (2) the intermediate series included, from front to back, the dorsal nucleus (anterior ventral focus of Nissl), the sensory nucleus (lateral focus of Kölliker and ventral focus of Nissl) with two satellite ganglia (semilunar nucleus or anterior skullcap and trapezoid nucleus or posterior skullcap), and the posterior or prebigeminal nucleus (lateral posterior nucleus of Nissl). Finally, (3) the internal series included, from back to front, the habenular ganglion, the internal nucleus (*nucleus medialis* of Kölliker and internal posterior nucleus of Nissl), the median or intermediate nucleus of Luys, the gray commissure ganglion or commissural ganglion, and the midline nucleus (*Mittellinie-Kern* of Kölliker) (Ramón y Cajal 1904).

## Development of the German School of thalamic studies: research in non-human primates by Cécile Vogt

In the first decades of the twentieth century, studies on the thalamus of humans and other mammals were fundamentally descriptive and comparative in nature. Each author used the parcellation scheme and terminology that seemed the best to name thalamic nuclei. For instance, the American neurosurgeon Ernest Sachs (1879–1958) reviewed the terminologies of Kölliker, Nissl, von Monakow, and Cajal for thalamic nuclei and used Burdach’s most simple scheme (Table 1):

“Recently, while in Professor Obersteiner’s laboratory in Vienna, I examined thirty-four mammalian brains (marsupials to man), comparing their optic thalami. This study, together with the brains of the cats and monkeys used in the present investigations, have led me to adopt, at Sir V. Horsley’s suggestion, the following classification of the thalamic nuclei until the precise connections of each part with the rest of the brain are determined. It corresponds very closely to the original description by Burdach [26], and at least for the results recorded below seems most practical:

Nucleus anterior.

Nucleus medius.

Nucleus lateralis (dorsal portion, middle portion, ventral portion)

Nucleus ventralis.

Centre median.

Nucleus arcuatus.

Pulvinar.” [Sachs (1909), pp. 100–101].

Sachs, under the supervision of Victor Horsley (1857–1916), carried out the first systematic study of thalamocortical and corticothalamic connections in cats and *Rhesus* macaques by means of the anterograde degeneration technique. He produced cortical or thalamic lesions in tens of animals using a stereotaxic surgery device (Sachs 1909). After a period of survival, animals were killed and their brains extracted, fixed, cut, and stained with Marchi’s technique for staining myelin of axons undergoing Wallerian degeneration (Marchi and Algeri 1886; Graybiel 1975). The major drawback of Marchi’s technique was that it could not detect degenerating axons without myelin sheaths, which are the dominant type of axons in terminal projection fields; thus, Marchi’s technique only allowed for gross tractography of projections. Sachs concluded that the thalamus of *Rhesus* macaques “must be regarded as consisting of an inner and outer division”; this outer division was the projection territory of the medial lemniscus, the superior cerebellar peduncle, and precentral motor areas (Sachs 1909).

**Fig. 5:**
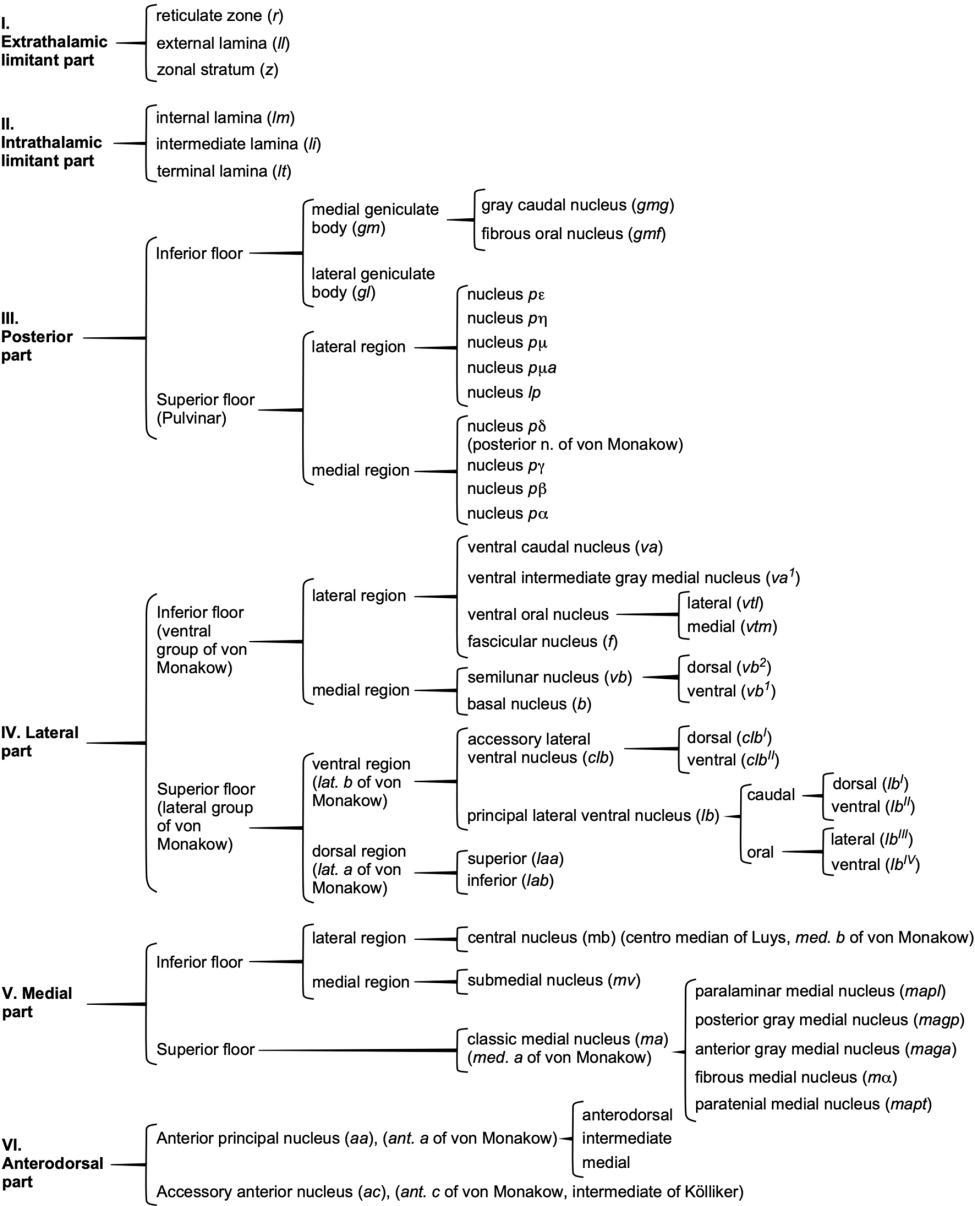
Parcellation and terminology of thalamic nuclei for a non-human primate species (*Cercopithecus mona*) by Cécile Vogt (1909)

Meanwhile, the French neuroanatomist Cécile Vogt (1875–1962) at the Neurobiology Institute of Berlin University carried out a careful description of thalamic myeloarchitecture in the non-human primate *Cercopithecus mona* and used Marchi’s technique to study thalamic connections in this species (Vogt 1909). In the introduction of her work, Cécile Vogt stated that, if anatomic divisions of the brain were to be made with physiological interest, it was mandatory to use the cytoarchitectural method (analysis of the size, distribution, and number of neuronal bodies in Nissl-stained sections), the myeloarchitectural method (analysis of myelinated axons in Weigert stained sections), and the fibrosystematic method (analysis of connections with Marchi’s and Gudden’s techniques). She divided the macaque thalamus into six portions including more than 40 nuclei and subnuclei. Four of these portions corresponded to nuclear groups that had been recognized since Burdach, and two corresponded to the fiber bundles that delimit and cross the thalamus. At the end of her article, Cécile Vogt included a summary table showing an excessively detailed classification and subclassification into portions, levels, and regions (Fig. 5).

Cécile Vogt, like other researchers of the Neurobiology Institute of Berlin University (Friedemann 1911), explained the correspondence between the thalamic nuclei of her parcellation scheme and that of von Monakow, reflecting the influence of the latter in the German School of thalamic studies.

## Systematization of thalamic parcellation and terminology of the German School by Rolf Hassler

As explained above, the seminal work by von Monakow (carmine red staining and Gudden’s technique in humans, dogs, and cats) was further developed by Cécile Vogt (myeloarchitecture with the Weigert technique, cytoarchitecture with the Nissl technique, and connections with the Marchi technique in non-human primates). The human thalamus was still unexplored with the newer and finer stains; accordingly, Cécile Vogt and her husband Oskar Vogt (1870–1959) took on the parcellation of the human thalamus:

*“Plus tard, nous avons pu, O. Vogt*^*4*^*) et moi, pousser plus loin la division du thalamus du chat ainsi que celle du thalamus de l*’*homme. Cette dernière n*’*a pas encore été publiée, bien que Monsieur Vogt l*’*ait déjà montrée au Congrès des psychiâtres allemands, à Jéna en 1903. Cette division était basée sur des différences myéloarchitecturales”.* [Later, O. Vogt and I have advanced the division of the thalamus of the cat as well as that of the human thalamus. The latter has not been published yet, although Mr. Vogt already presented it at the congress of German psychiatrists held in Jena in 1903. This division is based on myeloarchitecture] [Vogt (1909), p. 286].

At the height of the Second World War, when they were exiled to the Black Forest due to political discrepancies with the Nazi Government (Kreutzberg et al. 1992), the Vogts published their promised study on the myeloarchitecture of the human thalamus using the terminology that Cécile Vogt had used for the *Cercopithecus mona* (Vogt and Vogt 1941). This study was fundamental for Rolf Hassler (1914–1984), a disciple of Cécile and Oskar Vogt, to improve and systematize the parcellation and terminology that the Vogts applied to the thalamus of humans and *Cercopithecus mona*.

**Fig. 6:**
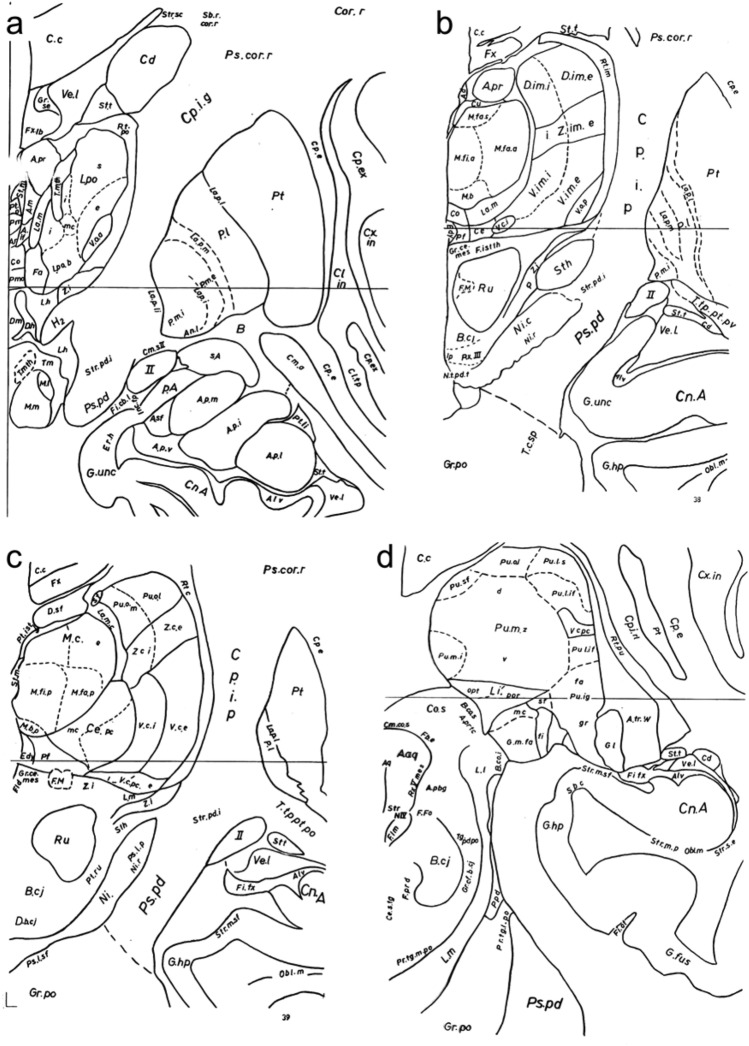
Drawings of coronal human brain sections depicting the thalamus from rostral (**a**) to caudal (**d**) by Hassler for the stereotaxic atlas of the human brain by Schaltenbrand and Bailey (1959). Abbreviations: A.if: N. Anteroinferior, A.m: N. Anteromedialis, A.pr: N. Anterior Principalis, A.r: N. Anteroreuniens, Ce: N. Centralis, Ce.mc: N. Centralis magnocellularis, Ce.pc: N. Centralis parvocellularis, Co: N. Commisuralis, Cu: N. Cucullaris, D.im.e: N. Dorso-intermedius externus, D.im.i: N. Dorso-intermedius internus, D.sf: N. Dorsalis superficialis, Edy: N. Endymalis, Fa: N. Fasciculosus, F.M: Fasciculus meynerti, G.l: Corpus Geniculatum Lateralis, G.m.fa: N. Geniculatus Medialis fasciculosus, G.m.fi: N. Geniculatus Medialis fibrosus, G.m.mc: N. Geniculatus Medialis magnocelularis, La.m: Lamella medialis, La.m.c: Lamella medialis caudalis, Li.opt: N. Limitans Opticus, Li.por: N. Limitans Portae, L.po: N. Lateropolaris, L.po.b: N. Lateropolaris basialis, L.po.e: N. Lateropolaris externus, L.po.i: N. Lateropolaris internus, L.po.s. N. Lateropolaris superior, L.po.mc: N. Lateropolaris magnocellularis, M.b: N. Medialis basialis, M.b.p: N. Medilais basialis posterior, M.c.e: N. Medialis Caudalis Externus, M.fa.a: N. Medialis Fasciculosus pars anterior, M.fa.p: N. Medialis Fasciculosus pars posterior, M.fa.s: N. Medialis Fasciculosus Superior, M.fi.a: N. Medalis Fibrosus pars anterior, M.fi.p: N. Medialis Fibrosus pars posterior, Pf: N. Parafascicularis, Pm: N. Paramedianum, Pm.o: N. Paramedianum oralis, Pt.o: N. Parataenialis oralis, Pt.ist: N. Parataenialis interstitialis, Pu.ig.fa: N. Pulvinaris intergeniculatus fasciculosus, Pu.ig.gr: N. Pulvinaris intergeniculatus griseus, Pu.l.if: N. Pulvinaris lateralis inferior, Pu.l.s: N. Pulvinaris lateralis superior, Pu.m.i: N. Pulvinaris medialis internus, Pu.m.z: N. Pulvinaris medialis zentralis, Pu.m.d: N. Pulvinaris medialis dorsalis, Pu.m.v: N. Pulvinaris medialis ventralis, Pu.o.l: N. Pulvinaris orolateralis, Pu.o.m: N. Pulvinaris oromedialis, Pu.sf: N. Pulvinaris superficialis, Rt.c: N. Reticulatus caudalis, Rt.im: N. Reticulatus intermedius, Rt.po: N. Reticulatus polaris, Rt.pu: N. Reticulatus pulvinaris, St.m: Stria medullaris thalami, St.t: Stria terminalis thalami, T.m.th: Tractus mammillothalamicus, V.c.e: N. Ventro-caudalis anterior externus, V.c.i: N. Ventro-caudalis anterior internus, V.c.pc: N. Ventro-caudalis parvocellularis, V.c.pc.e: N. Ventro-caudalis parvocellularis externus, V.c.pc.i: N. Ventro-caudalis parvocellularis internus, V.im.i: N. Ventro-intermedius internus, V.im.e: N. Ventro-intermedius externus, V.o.a: N. Ventro-oralis anterior, V.o.m: N. Ventro-oralis medialis, V.o.p: N. Ventro-oralis posterior, Z.i: Zona incerta, Z.c.e: N. Zentrolateralis caudalis externus, Z.c.i: N. Zentrolateralis caudalis internus, Z.im.e: N. Zentrolateralis intermedius externus, Z.im.i: N. Zentrolateralis intermedius internus. Reproduced, with permission from Thieme, from a copy of Schaltenbrand and Bailey (1959) in the Library of Universidad Autónoma de Madrid, Medical School

Hassler divided the human thalamus into cortical parts, which projected to the cerebral cortex, and stem–thalamic parts, which did not project to the cortex (Hassler 1959). The latter included the epithalamus, as well as midline (*Substantia Grisea Centralis Thalamica*) and intralaminar (*Involucrum Mediale*) nuclei. Hassler sorted the nuclei of cortical parts in different groups, namely: anterior, medial, medial geniculate, lateral geniculate, pulvinar, lateral, and reticular (Fig. 6).

His description of the lateral group nuclei was intended to be straightforward, but, obviously, he did not achieve this objective:

“Within the lateral nuclear mass there are ventral nuclei which receive extrathalamic fibers and which therefore are primarily receptive or sensory or cortical relay nuclei. Then there are dorsal nuclei which do not receive such extrathalamic fibers, and are therefore higher combining, integration or association nuclei. In the dorsal as well as the ventral nuclei, there are caudal, intemermedial, and oral nuclei (V. c, V. im, V. o, D. c, D. im, D. o). The nuclei ventrocaudales and ventroorales are both further subdivided in an anterior and a posterior part (V. c. p, V. c. a, V. o. p, V. o. a). In many nuclei, an internal or medial part is to be distinguished from an external or lateral part. That is to say, there is a V. c. e, a V. c. i, a V. im. e, a V. im. i, and a D. o. e, and a D. o. i”. [Hassler (1959), p. 251].

These and so forth, there were up to 27 subdivisions in the nuclear lateral mass. Altogether, Hassler increased the number of divisions and subdivisions of the human thalamus up to more than 100, which further aggravated the tendency to excessive parcellation that had already become apparent in the German literature since Cécile Vogt.

## The Anglo-American School of thalamic studies

In parallel to the development of the German School, several works were published by the Belgian psychiatrist Fernand D’Hollander (1878–1952), on the rabbit thalamus (D’Hollander 1913); by the American (born in Smyrna, Turkey) neurosurgeon Elisha Stephens Gurdjian (1900–1986), on the rat thalamus (Gurdjian 1927); and by the American (born in India) neuropsychiatrist David MacKenzie Rioch (1900–1985), on the cat and dog thalami (Rioch 1929). Based on the species they studied, D’Hollander, Gurdjian, and Rioch divided the thalamus into several broad groups (internal–middle–external; anterior–lateral–medial–midline–geniculate; and anterior–middle–midline–lateral–ventral–geniculate, respectively) that closely match the broad subdivisions of the early nuclear parcellations, including those of von Monakow and Kölliker (Table 1). Contrary to the complex German terminology developed by the Vogts and Hassler, these three authors did not categorize thalamic nuclei into level or region subdivisions. This terminology uses many terms that are familiar to us: anterior ventral, anterior dorsal, paracentral, medial dorsal, suprageniculate, etc. (D’Hollander 1913; Gurdjian 1927; Rioch 1929).

According to Jones (2007), the works of D’Hollander, Gurdjian, and Rioch were highly influential for researchers of the primate thalamus in Great Britain, Canada, and the USA. Their parcellations did not differ much from that by von Monakow, but their terminology was quite different from that of the German School. The most prominent Anglo-American authors who studied the cytoarchitecture, myeloarchitecture, and connections of thalamic nuclei in non-human primates were the British anatomist Wilfrid Edward Le Gros Clark (1895–1971), who developed his thalamic studies at Oxford University, and the Canadian neuroanatomist and neurosurgeon Arthur Earl Walker (1907–1995), who carried out his thalamic studies at the University of Chicago.

Le Gros Clark reviewed the knowledge available at that time on the structure and function of the thalamus of mammals (including humans) and compared morphological features and connections within the phylogenetic scale (Le Gros Clark 1932). He divided the thalamic nuclei into two categories, namely principal and intralaminar, with the principal nuclei classified as follows:

A. Thalamus lower levels:

1. Ventral group
2. Anterior group
3. Lateral geniculate
4. Medial geniculate

B.- Thalamus upper levels:

1. Lateral group
2. Centre median nucleus
3. Dorso-medial nucleus

Le Gros Clark also highlighted the great interest aroused in those days by the studies on the structure and connections of the brain stem and cerebral hemispheres, while studies on the thalamus were left aside:

“There are two main reasons for this neglect of thalamic anatomy. One is the confusion of nomenclature and the lack of definition which occur in the literature of the subject, and which have repeatedly led to misconceptions and misinterpretations which it has been impossible to harmonize with one another, and the other is the difficulty of applying the usual experimental methods to this part of the brain. It is clear, however, that the experimental method can be of little avail without a satisfactory definition of the elements which are to be subjected to study and, indeed, much of what little experimental work has hitherto been done on the thalamus has been vitiated by the neglect of a preliminary and detailed anatomical study. One of the main objects of this paper is to clear the terminological atmosphere so as to provide a sound anatomical basis for future investigations on the thalamic functions”. [Le Gros Clark (1932), p. 407].

The significance of the work by Le Gros Clark’s and his predecessors was acknowledged by the American anatomists Lester Ralph Aronson (1911–1996) and James Papez (1883–1958), who worked at Cornell University:

“The most recent works on the lower primates and closely related subprimate forms are those of Clark (1927 to 1932). They are of special significance since Clark, in his various writings, not only has followed the modern concepts concerning this region as developed by D’Hollander (1913), Gurdjian (1927), Rioch (1929) and others, but has also given considerable aid in solving many of the difficult problems concerning the thalamus.” [Aronson and Papez (1934), p. 27].

**Fig. 7:**
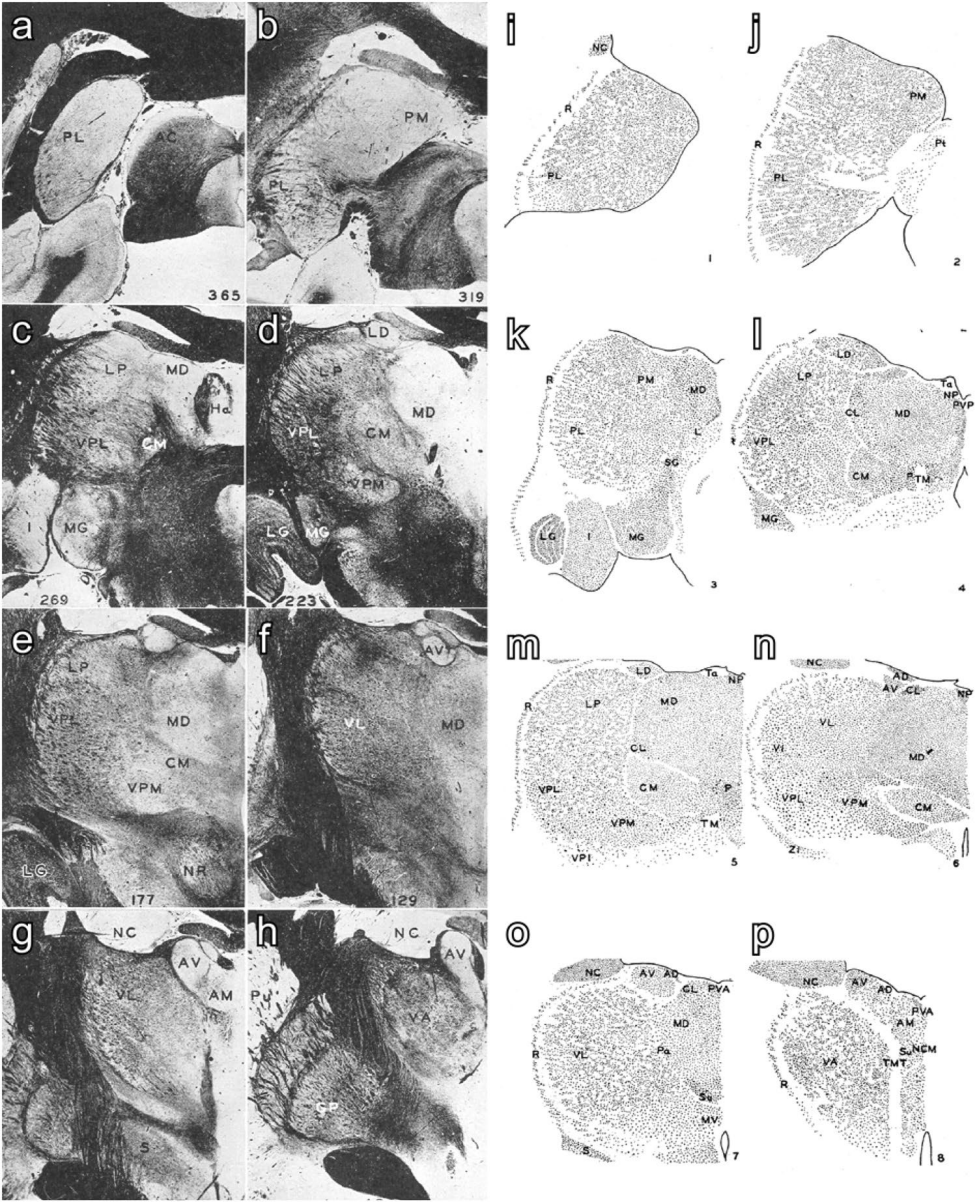
Parcellation and terminology of nuclei of the macaque thalamus by Walker (1938). **a**–**h** Micrographs of coronal sections of the macaque brain through the thalamus stained with the Pal-Weigert technique for myelin (these images were a courtesy of Professor B. Brower to Doctor Arthur E. Walker). **i**–**p** Camera lucida drawing from coronal sections of the macaque brain through the thalamus stained with the Nissl technique for cytoarchitecture. In both series, sections are arranged from posterior (**a**, **i**) to anterior (**h**, **p**). Abbreviations: AC: corpus cuadrigemina anterior, AD: nucleus anterodorsalis, AM: nucleus anteromedialis, AV: nucleus anteroventralis, CL: nucleus centralis lateralis, CM: nucleus centrum medianum, GP: globus pallidus, Ha: nucleus habenulae, I: nucleus pulvinaris inferior, L: nucleus limitans, LD: nucleus lateralis dorsalis, LG: corpus geniculatum laterale, LP: nucleus lateralis posterior, MD: nucleus medialis dorsalis, MG: corpus geniculatum mediale, MV: nucleus medialis ventralis, NCM: nucleus centralis medialis, NC: nucleus caudatus, NP: nucleus parataenialis, NR: nucleus ruber, P: nucleus parafascicularis, Pa: nucleus paracentralis, PL: nucleus pulvinaris lateralis, PM: nucleus pulvinaris medialis, Pt: pretectum, Pu: putamen, PVA: nucleus paraventricularis anterior, PVP: nucleus paraventricularis posterior, R: nucleus reticularis, S: corpus subthalamicum, Sg: nucleus suprageniculatus, Su: nucleus submedius, Ta: taenia thalami, TM: tractus meynerti, TMT: tractus mammillothalamicus, VA: nucleus ventralis anterior, VI: nucleus ventralis intermedius, VL: nucleus ventralis lateralis, VPI: nucleus ventralis posteroinferior,VPL: nucleus ventralis posterolateralis, VPM: nucleus ventralis posteromedialis, ZI: zona incerta. Reproduced, with permission from Chicago University Press, from a copy of Walker (1938) in the Library of Universidad Autónoma de Madrid, Medical School

Arthur Earl Walker’s monograph on the thalamus of non-human primates was published in 1938 (Fig. 7). It summarized the conclusions from both descriptive and experimental work carried out by the members of the Anglo-American School:

“Only a worker in research can know the pleasure of having a contemporary investigator in the same field, and I count myself fortunate in having had the advantage and stimulus of the successive papers on the thalamus by Professor Le Gros Clark and his school at Oxford”. [Walker (1938), p. X].

Furthermore, Walker’s monograph provided the following terminology:

I. Nuclear anterior group: *anterodorsalis*, *anteroventralis*, and *anteromedialis* nuclei.
II. Midline nuclei: *paratenialis*, *paraventricularis anterior*, *paraventricularis posterior*, *centralis medialis*, and *masa grisea centralis* nuclei.
III. Medial nuclei: *medialis dorsalis*, *centrum medianun* (*centre médian* of Luys), *submedius, medialis ventralis*, *parafascicularis*, *paracentralis*, and *centralis lateralis* nuclei.
IV. Nuclear lateral mass: *ventralis anterior*, *ventralis lateralis*, *lateralis dorsalis*, *lateralis posterior*, *ventralis posterior*, *ventralis intermedius*, *ventralis posteromedialis*, *ventralis posterolateralis*, *ventralis posteroinferior*, and *reticularis* nuclei.
V. Posterior nuclei: *pulvinaris* (*lateralis*, *medialis*, and *inferior*), *suprageniculatus, limitans*, *corpus geniculatum laterale pars dorsalis*, and *corpus geniculatum mediale* nuclei (Walker 1938).

**Fig. 8:**
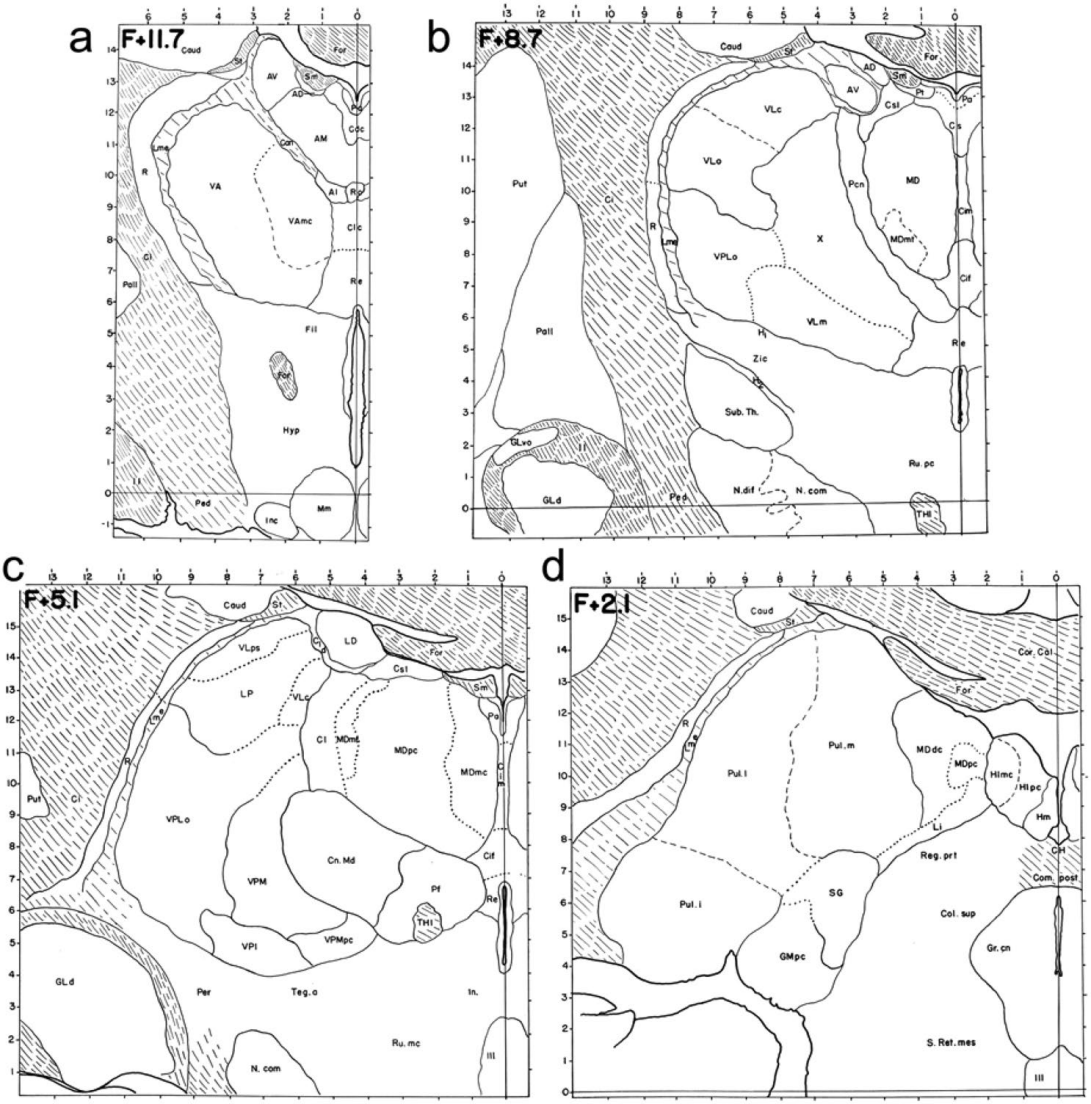
Schemes of the nuclear parcellation of the macaque thalamus by Olszewski (1952) drawn from coronal sections from rostral (**a**) to caudal (**d**), stained with techniques for cyto- and myeloarchitecture; stereotaxic coordinates are shown in the upper left corner of each scheme. Abbreviations: AD: N. anterodorsalis, AI: N. alaris, AM: N. anteromedialis, AV: N. anteroventralis, Can: capsule of the anterior nuclei, Cdc: N. centralis densocellularis, Cif: N. centralis inferior, Cim: N. centralis intermedialis, Cl: N. centralis lateralis, Clc: N. centralis latocellularis, Cld: capsule of the nucleus lateralis dorsalis, Cn. Md: N. centrum medianum, Cs: N. centralis superior, Csl: N. centralis superior lateralis, GLd: N. geniculatus lateralis dorsalis, GMpc: N. geniculatus medialis pars parvocellularis, Hlmc: N. habenularis lateralis pars magnocellularis, Hlpc: N. habenularis lateralis pars parvocellularis, Hm: N. habenularis medialis, LD: N. lateralis dorsalis, Li: N. limitans, Lme: lamella medullaris externa, LP: N. lateralis posterior, MD: N. medialis dorsalis, MDdc: N. medialis dorsalis pars densocellularis, MDmc: N. medialis dorsalis pars magnocellularis, MDmf: N. medialis dorsalis pars multiformis, MDpc: N. medialis dorsalis pars parvocellularis, Pa: N. paraventricularis, Pcn: N. paracentralis, Pf: N. parafascicularis, Pul. i: N. pulvinaris inferior, Pul. l: N. pulvinaris lateralis, Pul. m: N. pulvinaris medialis, Pt: N. parataenialis, R: N. reticularis, Re: N. reuniens, Ro: N. rotundus, Sg: N. suprageniculatus, sm: stria medullaris, st: stria terminalis, THI: tractus habenulo-interpeduncular, VA: N. ventralis anterior, VAmc: N. ventralis anterior pars magnocellularis, VLc: N. ventralis lateralis pars caudalis, VLm: N. ventralis lateralis pars medialis, VLps: N. ventralis lateralis pars postrema, VLo: N. ventralis lateralis pars oralis, VPI: N. ventralis posterior inferior, VPLo: N. ventralis posterior lateralis pars oralis, VPM: N. ventralis posterior medialis, VPMpc: N. ventralis posterior medialis, pars parvocellularis, X: area X, ZI: zona incerta. Reproduced, with permission from Karger Publishers, from a copy of Olszewski (1952) in the Library of Universidad Autónoma de Madrid, Medical School

Walker’s monograph inspired important works, such as the stereotaxic atlas of the macaque by the Lithuanian Jerzy Olszewski, (1913–1964), who worked in the Montreal Neurological Institute. Olszewski’s atlas is currently the most frequently used by researchers involved in the study of the macaque thalamus (Fig. 8; Olszewski 1952).

In the closing remarks of the atlas, the author comments on the values of cyto- and myeloarchitecture for thalamic parcellation and the meaning of divisions made by these methods:

“The value of architectonic methods lies in the fact that subdivisions based on these methods possess biological value. The delineated units have either different connection, or different ontogenetic development, or different reaction towards diseases, or a different cycle of involution. So any detection of architectonic difference points to a biological difference, without, however, specifying its nature–and, what is much more significant, its importance. Subdivisions based on architectural characteristics may be (and actually have been) advanced to very minute units. It seems, however, that such use of the method is rather undesirable, because anatomical subdivision, which is too far advanced ahead of other branches of neurobiology, is meaningless, uninteresting and uneconomical. For, whatever biological observation may be made, it will finally find its place in the complex edifice of science, but it would seem unwise to waste time and energy on elaboration of fine details so long as general gross features are still unknown”. [Olszewski (1952), p. 31].

In the above paragraph, there is a veiled criticism at the German School tendency for over-parcellation. Later on, Olszewski proposes a logic for meaningful thalamic parcellation:

“In accordance with the views expressed above, as the present work is purely morphological, no attempt has been made to group the described nuclei into units other than those generally accepted. When the existing and recognized subdivisions were found to be insufficient, and striking morphological differences forced recognition of new units, these have been held within the limits of nuclei recognized by previous authors, preference being given to Walker’s subdivision. Walker’s studies furnished a good example of how the functional approach influences and dominates purely morphological investigations. The thalamic subdivisions which existed when Walker began his studies were more elaborate and more detailed than the subdivision which resulted finally from Walker’s investigations. However, none of the other subdivisions has gained such a wide recognition as that of Walker. This recognition is due to the fact that Walker gave to his nuclei a functional meaning. As the connections and the function of a given nucleus, and not its appearance under the microscope, was the chief interest for most of the neurobiologists, it is clear that Walker’s approach has received more attention and has been more readily accepted than purely morphological studies”. [Olszewski (1952), pp. 31–32].

These paragraphs from the Atlas of Olszewski contain an epistemological and ontological formulation for neuroanatomical research which is commonly known as Functional Neuroanatomy. According to Olszewski, neuroanatomy should provide divisions of the brain into units that have functional meaning. He also mentions that brain units delineated by cyto- and myeloarchitecture also have different developmental origins, different vulnerability to diseases, and different aging processes. He concludes that the definitive solution for thalamic parcellations and terminology:

“… will be the introduction of completely new names, which should be based not on topographical or cell structure criteria, but on the connections and function”. [Olszewski (1952), p. 32].

The prestige of Walker’s approach, as highlighted by Olszewski, was acknowledged through Walker’s participation in the stereotaxic atlas of the human brain by Georges Schaltenbrand and Percival Bailey (1959). In this atlas, Walker wrote the chapter on thalamus physiology and pathophysiology with extensive discussion of thalamocortical connections. In contrast, the chapter dealing with the anatomy of the human thalamus was written by Rolf Hassler, who used a nomenclature drastically different to Walker’s.

## German and Anglo-American Schools of thalamic studies face to face

The nuclear parcellation of the primate thalamus of the German School, systematized by Hassler, relies on myeloarchitecture and cytoarchitecture, is essentially morphological, divides the thalamus into more than 100 nuclear subdivisions that often lack functional meaning, and uses complex terms difficult to remember. The pioneering texts of the German School were published in French and German, so many contemporary neuroscientists cannot understand them. Nevertheless, because this nomenclature was included in one of the twentieth century’s most widely disseminated stereotaxic atlases (Schaltenbrand and Bailey 1959), it has become familiar to neurologists and neurosurgeons all over the world. Notably, the terms referring to motor ventral nuclei in the human thalamus are widely used in stereotaxic surgery for thalamotomy (e.g., Oh and Park 2021).

The German terminology has also been used, with some modifications, in other atlases for human stereotaxic surgery (Andrew and Watkins 1968; Van Buren and Borke 1972). Strikingly, Walker wrote the prologue for these atlases. Also, the German terminology was the basis for the terminology proposed by the symposium organized by the International Brain Research Organization held in Louvain in 1963 (“The Methodology of the Morphological Analysis of Human Thalamus”). The aim of this symposium, in which most participants were European scientists, was to reach “a definitive agreement” on thalamic parcellation and terminology. However, the resulting consensus on parcellation and terminology (Dewulf 1971) presented variants of the German School and did not reach the mainstream of thalamic studies.

The Anglo-American parcellation, systematized by Walker, is mainly based on comparative and experimental studies of the thalamus of different mammals including non-human primates. This parcellation consists of approximately 50 nuclei and subnuclei, most of which are functionally significant because their definition relies on large amounts of data from experimental neuronal tracing studies. In addition, the terms used by the Anglo-American School to name nuclear divisions are more systematic and easier to memorize than those of the German School. Finally, the Anglo-American School has been developed by English-speaking scientists writing in English, which has made their articles more easily accessible to researchers all over the world. The Anglo-American parcellation and its associated terminology are extensively used by neuroscientists and experimental researchers, mostly for studies on non-human primates, in part due to the usefulness of Olszewski’s atlas (1952). The Anglo-American parcellation has also been used for studies on the human thalamus (Sheps 1945; Toncray and Krieg 1946; Dekaban 1953; Kuhlenbeck 1954; Van Buren and Yakovlev 1959; Hirai and Jones 1989), although only one relevant atlas for stereotaxic surgery uses it (Talairach and Tournoux 1988).

What did Hassler and Walker, the most outstanding representatives of their respective schools, think about the “terminological atmosphere” mentioned by Le Gros Clark in 1932, an atmosphere that they both still breathed? Hassler and Walker themselves answered the above question in the chapters they wrote for Schaltenbrand and Bailey’s atlas:

Hassler: “(…) since Meynert (1872) and v. Monakow (1895) one distinguishes between ventral and dorsal nuclei of the lateral nuclear mass. To call only the latter ones lateral nuclei is not very useful because the ventral nuclei are also lateral and belong to the lateral nuclear mass. In this nuclear mass the names which the English literature uses are illogical, so that they are difficult to learn and cannot be extended any further. The most unfortunate is the name VL (ventral lateral), which, according to Walker (1938) and recently according to Olszewski (1952) should embrace large parts of the dorsal nuclei in spite of the fact that it is only a rostral ventral nucleus which sometimes is distinguished from the VPL and sometimes from the VA or AV, where this AV sometimes even should have nothing to do with the anteroventralis (AV) of the anterior nuclear mass. How can anybody who is not specialized in this region find his way about in this nomenclature which is not really thought out, not systematically ordered, and not capable of further development? It is necessary to do without some of these names in order to introduce a nomenclature which is systematically built up. Such a nomenclature has been proposed by C. and O. Vogt (1941) and has been further developed by myself”. [Hassler (1959), p. 251].

Walker: “As soon as neuroanatomists began comparative studies, they realized that the broad divisions of the thalamus recognized by clinicians were quite inadequate for detailed cytological and physiological studies. Then began a series of attempts to designate thalamic components by topographic or descriptive adjectives, numbers and letters, both Greek and Arabic. From this confusion has come isolationism – each school of thalamic thought rigidly adhering to its own code and ignoring all others. This schizophrenic reaction has made thalamic studies difficult and is largely ignored by clinicians who feel frustrated when they encounter such terms as nucleus ventro-caudalis posterior internus.

Just criticism may be leveled at all present thalamic terminologies. It is time that the entire subject was reviewed and a simple international nomenclature agreed upon. Since present classifications of thalamic nuclei are partly based on morphological and partly on physiological factors, which are poorly stablished and controversial, all which attempt a precise delimitation have inherent the uncertainty of the functional aspects of their subdivisions. Because research is changing the concepts of the thalamic and cortical physiology so rapidly, it seems to the author, advisable to retain the present thalamic terminology with all its defects until neuroanatomists and neurophysiologists have plotted the diencephalon more precisely”. [Walker (1959), p. 291-292].

It seems that the “terminological atmosphere” was fairly strained at the end of the 50s.

## Parcellation of the primate thalamus after Walker and Hassler

The 50s, 60s, and 70s of the twentieth century were a period for technological innovation in neuroanatomy laboratories. Modern neuroscience was emerging. New histoenzymatic stains (like acetylcholinesterase), immunohistochemistry, and in situ hybridization allowed for precise localization of enzymatic activities, expression of proteins, and transcription of genes in brain tissue and nerve cells. These techniques provided neuroanatomists with much finer tools than cyto- and myeloarchitectonic stains to study the structure of the brain and its cells. Also, the tracing technique developed by the Dutch neuroanatomist Walle Nauta (1916–1994) could detect non-myelinated degenerated axons and markedly improved on the results of Marchi’s technique. Finally, modern tract-tracing techniques based on the axonal transport of various tracers (e.g., tritiated amino acids, horseradish peroxidase, and fluorescent dyes) provided more precise data on synaptic connections than the old techniques based on secondary degeneration (Cowan 1998; Cowan et al. 2000).

The combined use of the new techniques significantly changed the understanding of thalamic structure and function in the second half of the twentieth century and also had an impact on nuclear parcellation. The most relevant researcher on thalamic structure of this period was the American (born in New Zealand) neuroanatomist Edward G. Jones (1939–2011), who proposed a parcellation and nomenclature for the primate thalamus based on the Anglo-American School, especially on the works of Walker and Olszewski. The aim was to name the thalamic nuclei of all primate species (Jones 1985, 2007; Hirai and Jones 1989). In developing such a nomenclature, Jones used chemoarchitectural (acetylcholinesterase technique), cytoarchitectural (Nissl technique) and, to a lesser extent, myeloarchitectural criteria. Jones also took into consideration the projection territories of subcortical structures onto the thalamus (the fibrosystematic method of Cécile Vogt) studied with modern tracers. The parcellation and terminology of Jones has the advantage of being simple and readily applicable to the thalamus of all primate species. Its main drawback lies in the parcellation of the motor thalamus, which is less accurate than the one proposed by Olszewski. Thus, Jones suggested including the entire cerebellar projection territory into a single large nucleus (ventral lateral posterior, VLp) with no attention to the topographic arrangement of cerebellar axons (Asanuma et al. 1983a; Asanuma et al. 1983b, c). The major target of stereotaxic surgery in the thalamus (Vim nucleus in Walker and V. im nucleus in Hassler) was included in the VLp nucleus of Jones (Macchi and Jones 1997). In contrast, the targets of thalamotomy were described more precisely in the parcellation proposed by Hassler (1959). Importantly, Jones never provided stereotaxic coordinates for his parcellation: therefore, his nomenclature is not helpful for neurosurgeons performing thalamotomies. Table 2 summarizes the equivalences of terms for the human thalamic nuclei in the Hassler and Jones parcellations.

**Table 2:**
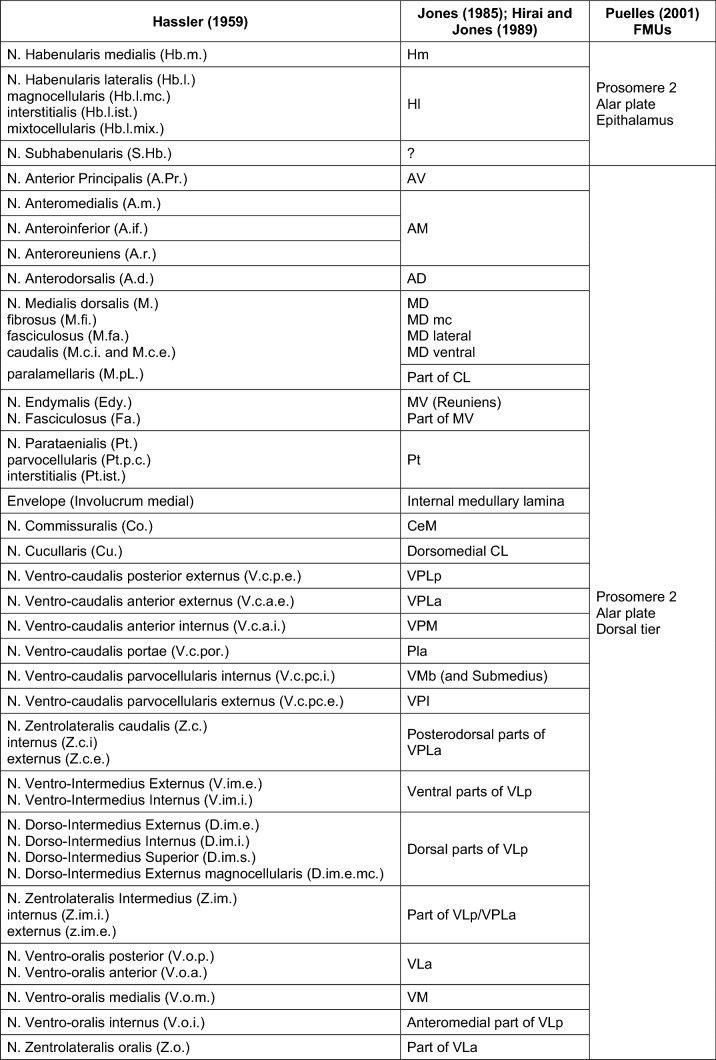

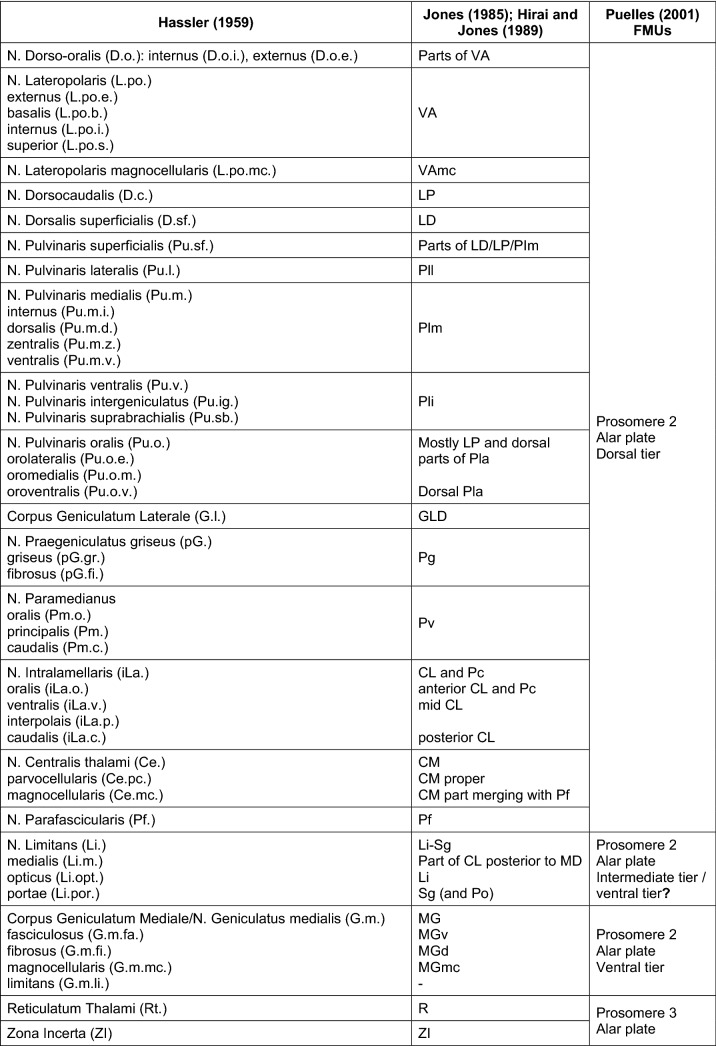
Comparison of human thalamus parcellations with ontogenetic units (fundamental  morphological units, FMUs)

Jones’ studies promoted the thalamic parcellation and terminology of the Anglo-American School. Since 1970, Olszewski’s terminology was used in most experimental studies of thalamic structure and connections in non-human primates (e.g., Kievit and Kuypers 1977; Schell and Strick 1984; Goldman-Rakic and Porrino 1985; Selemon and Goldman-Rakic 1988; Darian-Smith et al. 1990; Barbas et al. 1991; Sánchez-González et al. 2005). In this period, alternative parcellations of the thalamus of macaques (e.g., Ilinsky and Kultas-Ilinsky 1987) were also based on Olszewski’s scheme. Jones’ terminology was also used in experimental and neuropathological studies on the human thalamus (e.g., Halliday et al. 2005; García-Cabezas et al. 2007). It is of note that some authors using the scheme of the Anglo-American School rejected the simplification of the Ventral Lateral Posterior region into one single nucleus as proposed by Jones and divided the VLp into several nuclei and subnuclei (Stepniewska et al. 1994; Morel et al. 2005).

It is noteworthy that, in spite of all the experimental data obtained from macaques using the schemes of the Anglo-American School since 1980, at the end of the twentieth century the “terminological atmosphere” was still not totally clear and the German terminology and parcellation were still being used. An example of mixed use of both terminologies is the book “Thalamus”, edited by Mircea Steriade, David A. McCormick and Jones himself in 1997. In Chapter 9 of Volume II (“A description of the human thalamus”), the neuroanatomist Jones utilizes the nomenclature put forward by Hirai and himself (Jones 1997). In contrast, in Chapter 11 of Volume II (“Functional organization of the human thalamus: stereotaxic interventions”), the Japanese neurosurgeon Chihiro Ohye turns to Hassler’s 1959 nomenclature (Ohye 1997), while in Chapter 12 of Volume II (“Thalamic functions as interpreted from human lesions”) the Italian neurologist Giorgio Macchi uses the nomenclature proposed by Jones (Macchi 1997). In Chapter 13 of Volume II (“Pain processing in the human thalamus”), Frederick A. Lenz and Patrick M. Dougherty, neurosurgeon and neuroscientist, respectively, use Jones’ nomenclature (Lenz and Dougherty 1997). Finally, in Chapter 14 of Volume II (“Degenerative diseases of the human thalamus”), the Belgian neuropathologist Jean Jacques Martin uses the consensus nomenclature of 1971 (Martin 1997).

At present, the preponderance of the parcellation and terminology for thalamic nuclei developed by the Anglo-American School is near to total in experimental studies on non-human primates. Jones’s parcellation and terminology for the human thalamic nuclei are used in contemporary text books of human anatomy and neuroanatomy (Nieuwenhuys et al. 2008; Standring 2020) and have replaced Hassler’s schemes in several contemporary stereotaxic atlases of the human brain, either totally (Morel et al. 1997; Niemann et al. 2000) or partially (Mai et al. 2004). The use of other terminologies (e.g., Percheron et al. 1996; Percheron 2004; Ilinsky et al. 2018) has not spread beyond the works of their proponents.

The above instances depict a landscape characterized by the potential final vanishing of the German School schemes, but the parcellation and terminology of Hassler still remain in the old atlases of the human thalamus used in the clinical settings of neurosurgery and neuroimaging. Mai and Majtanik (2018) have made the latest attempt to overcome the problems in thalamic research caused by using different parcellations and terminologies for nuclei of the human thalamus. The approach of these authors differs from those used in classic parcellation studies. They created a normalized thalamus using the open Human Brain Atlas of the Montreal Neurological Institute. Then, they reconstructed in 3D the nuclear parcellation from nine neuroanatomical atlases of the human thalamus (in 2D) within their normalized 3D thalamus. Their next step was to evaluate consistencies and differences in the delineation of thalamic nuclei across atlases within the same standard frame. Finally, they produced a multilayered terminology for each thalamic nucleus depending on the level of concordance across the nine atlases. Thus, they do not assign “true” names to each site in the human thalamus for potential electrical stimulation; rather, they provide to each thalamic site several terms from the terminologies of the nine atlases with their corresponding parcellations across the atlases. This alternative approach aims to convert terminology for thalamic nuclei into spatial coordinates, which may be a practical tool for thalamic surgery, and could complement classical approaches based on architecture and connections as well as the new genoarchitectonic studies that will be commented in the following section.

## Genoarchitectonic studies and the New Neuromorphology: an alternative paradigm for thalamic parcellation

The preceding paragraphs evidence that advances in thalamic parcellation, terminology, structure, and function developed in parallel to technological and methodological advances. The magnifying lens of Burdach revealed several nuclei in the human thalamus. Transparent sections, carmine red, Nissl, and Weigert stains allowed the identification of more nuclei, but did not provide information on connections and, consequently, hindered insights into thalamic function. Gudden and Marchi’s techniques used by von Monakow, Sachs, Cécile Vogt and Walker showed that the thalamus was the projection target of subcortical pathways, like the medial lemniscus and the superior cerebellar peduncle, and was reciprocally connected with the cerebral cortex. Even though secondary degeneration was highly variable and difficult to interpret, those early tract-tracing techniques provided a broad picture of thalamic connectivity that gave functional insight into the thalamic parcellation of the Anglo-American School. More recently, starting in the second half of the twentieth century, modern neural tracers and modern stains have increased and refined our knowledge of thalamic structure and connections.

As already mentioned, one of the reasons for the success of the parcellation and nuclear terminology of the Anglo-American School of thalamic studies over the German School is the functional (connectional) approach taken by Anglo-American researchers in their morphological investigations; it enabled them to infer functional meaning for their nuclei. The thinking of the Anglo-American School is represented by Olszewski’s statement that the definitive parcellation and terminology of thalamic nuclei would involve the introduction of completely new terms based on connections and functions (Olszewski 1952). For Olszewski and many other neuroanatomists of the twentieth century (e.g., Brodal 1981), the ultimate goal of anatomical studies was to provide insight into function to help clinicians in diagnosis and in finding treatments for neurological diseases, like thalamotomy for tremor. This approach, known as Functional Neuroanatomy, is based on the structural study of the brains of adult humans and experimental animals and aims at elucidating synaptic circuits and connections to explain function and clinical symptoms. Precise knowledge of connections and synaptic circuits is needed to correlate anatomical findings with functional studies (including stimulation studies, electrophysiological recordings, and optogenetics), and with changes in behavior after brain lesions in non-human primates and humans (Fuster 1980; Kim et al. 2017). The integration of all these structural and functional techniques and approaches have contributed to Functional Neuroanatomy and to the refinement of thalamic parcellations.

The terms and descriptors used to name brain structures in Functional Neuroanatomy are topographical: they describe the anatomical positions and relations of thalamic nuclei in relation to the axes of the adult brain. Thus, in the thalamus there are ventral nuclei, dorsal nuclei, anterior nuclei, etc. Other terms refer to the shape of brain structures, like geniculate (knee) nuclei. Most neuroanatomy textbooks are written within the paradigm of Functional Neuroanatomy (e.g., House and Pansky 1960; Heimer 1983; Afifi and Bergman 2005; Haines 2015) and topographical descriptors for brain structures are widely used in clinical practice.

An alternative approach to Functional Neuroanatomy, not based on the relation between structure and function, but rather on the ontogeny and phylogeny of brain structures, has been advocated since the beginning of neuroscience. Santiago Ramón y Cajal may have been the first in calling attention to the conceptual value of embryology:

“En realidad, la disposición de una neurona adulta representa el término de una serie de movimientos, de impulsos interiores y exteriores, que obraron durante la época embrionaria y juvenil, y cuya puntual determinación constituirá, andando el tiempo, la verdadera explicación de la organización celular. La razón de la forma está, pues, por entero en la función actual ó pasada”. [In reality, the disposition of an adult neuron represents the last in a series of movements, of inner and outer impulses, at work during the embryonic and juvenile period, and whose precise determination will constitute, in time, the true explanation of cellular organization. The reason for form thus lies entirely in present or past function] [Ramón y Cajal (1899), p. VIII]

This idea of Cajal has been shared by many neuroscientists in the twentieth century who devoted time and energy to unveiling the developmental mechanisms of the nervous system (e.g., Hamburger 1989). But only in the last decades has the paradigm of Functional Neuroanatomy been challenged by an alternative paradigm rooted in evolutionary developmental biology. This alternative paradigm, which has been called New Neuromorphology (Nieuwenhuys and Puelles 2016), was possible thanks to the use of in situ hybridization techniques on embryonic tissue to detect the expression of gene transcripts in the course of development in individuals from different animal species. If classic Nissl and Weigert stainings provide pictures of cyto- and myeloarchitecture and modern histoenzymatic techniques and immunohistochemistry provide pictures of chemical architecture, in situ hybridization in embryonic tissue provides pictures of genoarchitecture (Puelles and Ferran 2012). Applied to developing neuronal systems, genoarchitecture displays patterns of organization with ontogenetic and phylogenetic significance.

**Fig. 9:**
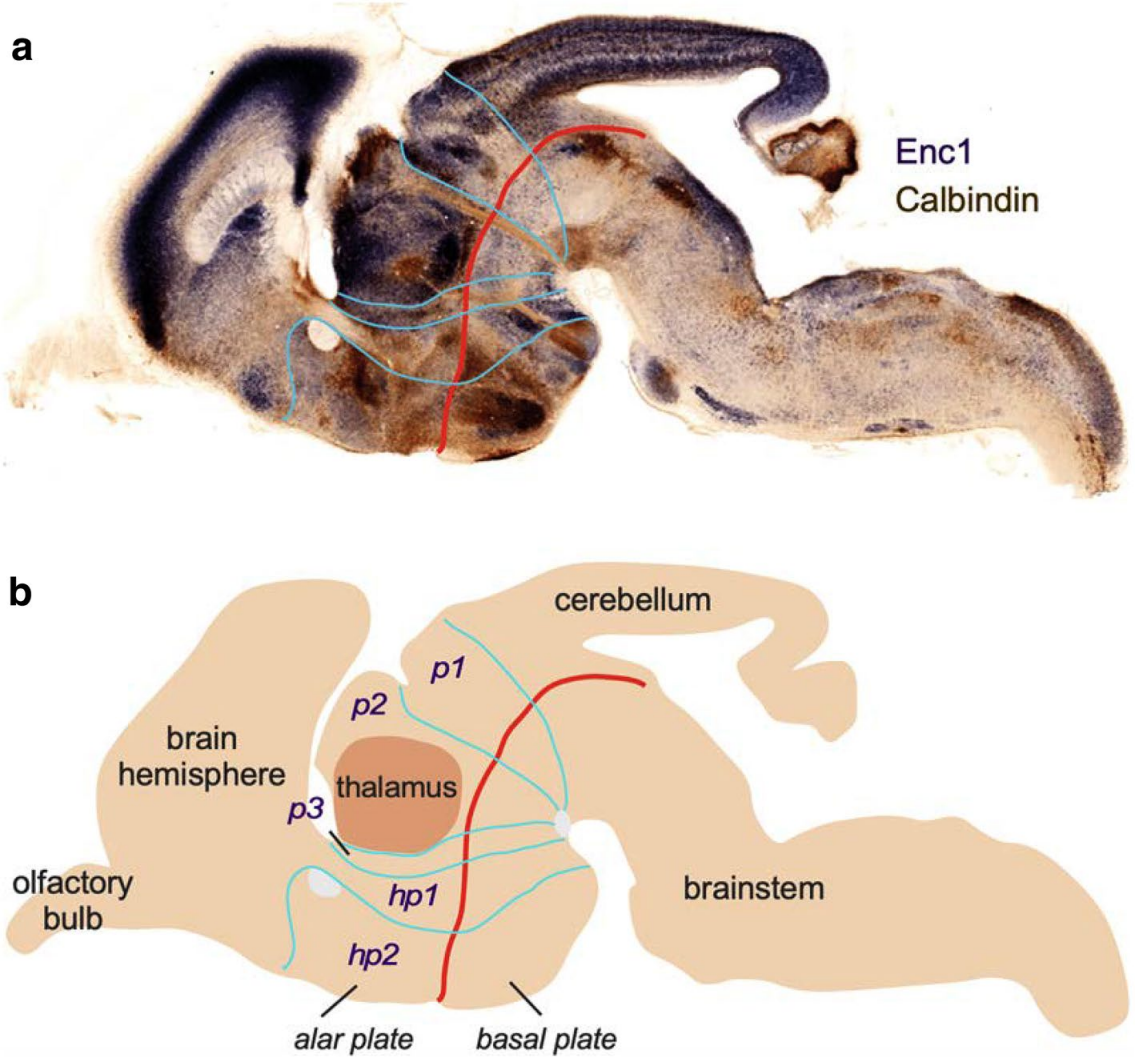
Fundamental morphological units (FMUs) giving rise to the diencephalon and telencephalon. **a** Parasagittal section of a mouse central nervous system at embryonic age E18.5 processed by in situ hybridization for Enc1 (Ectoderm-neural cortex protein 1 gene; blue color in the micrograph) and immunohistochemistry for the calcium-binding protein Calbindin (brown color in the micrograph). This figure is a courtesy of Doctors Luis Puelles and Margaret Martínez-de-la-Torre. **b** Diagram depicting the FMUs giving rise to the diencephalon and the telencephalon; FMUs are delimited by the intersection of transverse units [prosomeres 1–3 (p1–p3) and hypothalamic prosomeres 1 and 2 (hp1 & hp2)] with alar and basal plates. The blue lines mark the boundaries of the prosencephalic neuromeres. The red line marks the alar–basal plate boundary. The FMU that gives rise to most thalamic nuclei is in the alar plate of p2, while the reticular nucleus is derived from p3

The major proponent for the New Neuromorphology is the Spanish embryologist Luis Puelles (1948-), who has studied, using in situ hybridization, the spatial expression of morphogenetic genes in the neural plate and early neural tube across development in vertebrate embryos (Puelles and Rubenstein 1993; Puelles 2018; García-Calero and Puelles 2020). The New Neuromorphology defines brain structures according to their embryological origin as fundamental morphological units (FMUs). These units are blocks in the neural plate and wall of the early neural tube that are specified by the inductive effects of morphogenetic proteins secreted by organizers. The expression of morphogenetic genes delineates boundaries in the neural plate and early neural tube that delimit FMUs (Fig. 9); according to the New Neuromorphology, those boundaries can be considered as “natural” boundaries in the nervous system, and FMUs the natural “bricks” for building brains. Actually, the studies by Puelles and his colleagues show that the neural tube of all vertebrate species is built according to a “bauplan” (building plan) composed of the same set of FMUs for fish, amphibia, reptiles, birds, and mammals.

The primary events of specification are followed by the secondary events of histogenesis (cell proliferation, cell migration, axon navigation, cytoplasmic differentiation, synapse formation, synapse pruning, myelination, etc.) that will occur in each FMU. Differences in magnitude and time course of the secondary histogenesis events across FMUs result in differential growth and give rise to the tertiary events of morphogenesis, such as focal thickening of the wall of the neural tube, the appearance of flexures and sulci, and the formation of grisea like thalamic nuclei (Puelles and Ferran 2012; Puelles 2018; García-Calero and Puelles 2020). Therefore, while Functional Anatomy defines brain structures and their anatomical relations after tertiary events have shaped the nervous system, the New Neuromorphology defines brain structure based on the causal mechanisms that specify FMUs. Thus, Functional Neuroanatomy and the New Neuromorphology are based on different epistemological grounds (tertiary versus primary events) with different ontological values (nuclei versus progenitor domains). These epistemological and ontological differences are reflected in the anatomical descriptors and terms used by each of the two paradigms. Terms that name brain structures in the scope of the New Neuromorphology are based on the invariant topological positions of FMUs in relation to the axes of the neural plate and early neural tube (Puelles and Rubenstein 2015; Puelles 2018; García-Calero and Puelles 2020); these descriptors do not necessarily coincide with the topographical descriptors observed in the adult brain that are used in the scope of Functional Neuroanatomy (Puelles 2019).

The New Neuromorphology has redefined the developmental origin of the thalamus, which was considered part of the diencephalon together with the hypothalamus. The genoarchitectonic studies of Puelles and his colleagues have shown that the diencephalic vesicle is not a single developmental unit. Rather, the diencephalon is composed of several FMUs, whose different magnitudes in secondary histogenetic events cause focal thickening of the neural tube in the shape of a vesicle (Puelles and Rubenstein 1993, 2015; Martinez-Ferre and Martinez 2012). Knowledge of the FMUs that give rise to the thalamus across vertebrate species is still incomplete, but it has been demonstrated that they are in the alar plate of prosomere 2, except for the reticular nucleus, which derives from the alar plate of prosomere 3 (Fig. 9). There are four FMUs in the alar plate of prosomere 2, which are called anteroventral tier, ventral tier, intermediate tier, and dorsal tier. The medial geniculate nucleus is derived from the ventral tier, the posterior nucleus (nucleus rotundus in reptiles and birds) is derived from the intermediate tier, and the lateral geniculate with all the other thalamic nuclei are derived from the dorsal tier (Redies et al. 2000; Puelles 2001).

More data on the primary events of FMUs’ specification in the alar plate of prosomere 2 are needed to better understand the ontogeny and phylogeny of the thalamic nuclei. Once these data are available, it will be possible to parcellate thalamic nuclei in adult primates tracing each nucleus back to its corresponding FMU, because, in spite of any morphogenetic changes, the invariant topological relations of FMU derivatives can be identified in adult brains (Table 2). It has been possible to do this in the hypothalamus of adult *Rhesus* macaques (Wells et al. 2020), because there is abundant data on the FMUs that give rise to the mammalian hypothalamus. Genoarchitectonic studies in adult animals using data on the expression of thousands of genes at the level of thalamic nuclei (Nagalski et al. 2016) or neurons (Phillips et al. 2019) will also impact future thalamic parcellations and terminologies. The novel techniques of spatial transcriptomics (Stahl et al. 2016; Vickovic et al. 2019; Stickels et al. 2021) should identify the expression of thousands of genes in intact brain tissue from non-human primates and humans and will likely help in understanding species similarities and differences, as well as in refining the boundaries between thalamic nuclei.

## Conclusion

Understanding the logic underlying thalamic layout, in particular its parcellation in different nuclei, has been a challenge for the last two centuries and is still an unresolved question. The thalamic parcellations proposed over the years have sprung up in parallel to technological advances that enabled the emergence of novel viewpoints with different epistemological premises and ontological consequences. These paradigms permitted a progressive understanding of thalamic structure and function from different points of view. This history is summarized in Table 3 and expounded in detail in the preceding sections.

**Table 3:**
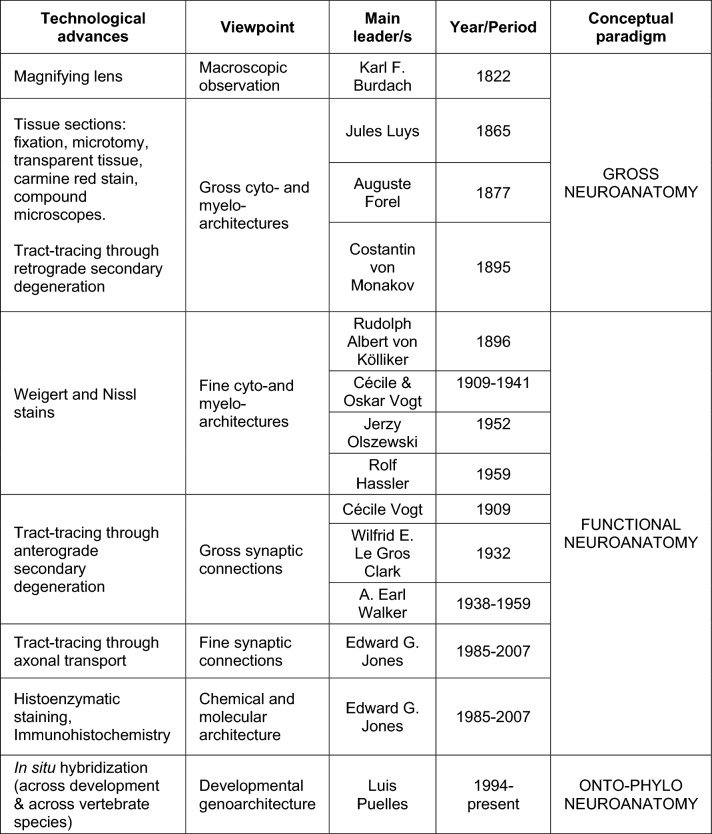
Parallel events in the history of primate thalamic parcellation

Until recently, the paradigm of Functional Neuroanatomy has prevailed, but at the beginning of the twenty-first century a new paradigm to explain the building logic of the brain is available: the so-called New Neuromorphology (Nieuwenhuys and Puelles 2016). We propose a more explicative name for this paradigm: Onto-Phylo Neuroanatomy. Onto-Phylo Neuroanatomy relies on identifying the invariant and differential expression of genes in particular territories of the developing neural plate and tube across species. It is a novel viewpoint worth exploring and developing in the twenty-first century. Onto-Phylo Neuroanatomy should allow sound comparative studies on the cellular, connectional, genetic, and molecular architectures of the thalamic nuclei across species. In the end, it should help clarify interspecific similarities and differences, including those between the human thalamus and that of other primates and mammals and allow the proposal of homology hypotheses for thalamic nuclei among these species (Puelles and Medina 2002).

Conceivably, in a few decades, our understanding of the thalamus, in particular its functions and relationships with other brain regions, will be enriched and novel challenges will emerge. We believe that both paradigms, Functional and Onto-Phylo Neuroanatomy, reveal different aspects of the nervous system and will complement, rather than exclude each other. Thalamic parcellations and terminologies of the future will likely rely on both.

## Data Availability

Does not apply. This is a review on the historical evolution of techniques and concepts in thalamic nuclei parcellation. We do not have data that should be made available.
